# Multiplexed Tandem Mass Spectrometry Imaging Enables Large‐Scale Isomer Mapping and Annotation in Tissues

**DOI:** 10.1002/anie.202522119

**Published:** 2026-03-30

**Authors:** Varun V. Sharma, Gabor Toth, Robert Martinis, Cathrin E. Hansen, Gijs Kooij, Ingela Lanekoff

**Affiliations:** ^1^ Department of Chemistry for Life Science Uppsala University Uppsala Sweden; ^2^ Center of Excellence for the Chemical Mechanisms of Life Uppsala University Uppsala Sweden; ^3^ Department of Molecular Cell Biology and Immunology Amsterdam UMC Location VU Medical Center Amsterdam the Netherlands; ^4^ Amsterdam Neuroscience Amsterdam UMC Amsterdam the Netherlands; ^5^ MS Center Amsterdam Amsterdam UMC Location VU Medical Center Amsterdam the Netherlands; ^6^ Amsterdam Institute for Immunology and Infectious Diseases Amsterdam UMC Amsterdam the Netherlands

**Keywords:** annotation, isomers, lipids, mass spectrometry imaging, multiple sclerosis

## Abstract

Accurate molecular annotation is essential for deciphering biochemical processes in spatial biology. Here, we present a scalable and broadly applicable molecular annotation tool for tandem mass spectrometry imaging (MS^2^I). Our workflow includes parallel image acquisition (PIA) for parallel MS^2^I and an open‐access computational framework for spatial similarity networking (SSN) that enables molecular annotation of MS^2^I data with isomeric specificity. The PIA enables simultaneous untargeted MSI and targeted MS^2^I ensuring structure‐specific imaging of hundreds of molecules in a single experiment. The SSN increases annotation confidence through graph‐based spatial correlation of product ion distributions, opening up new avenues for data investigation and annotation from both MSI and MS^2^I data. By integrating PIA and SSN into a single workflow, we visualize and annotate 134 phospholipid isomers and isobars in mouse brain tissue. Furthermore, we demonstrate the biological utility of the platform by mapping cholesterol metabolism in human multiple sclerosis brain tissue, revealing lesion‐associated cholesterol oxidation pathways. Finally, we propose annotation confidence levels for structural annotation in MSI. Overall, PIA and SSN together provide large‐scale, structure‐specific MSI, expanding the scope for spatial metabolomics, lipidomics, and chemical pathology through molecular annotation beyond current capabilities.

## Graphical abstract

Parallel mass spectrometry imaging acquires high mass resolution and tandem mass spectrometry images from 108 isolation windows in a single run. Spatial similarity networking clusters product ions by spatial distribution, enabling annotation of isomers and isobars as demonstrated for phospholipids and oxidized cholesterol in mouse and human brain tissue.

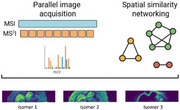

## 1 Introduction

The structures of small molecules that regulate cellular processes are highly specific, with isomeric forms often generating distinct biological effects [1]. Structural isomers of metabolites and lipids can alter the function of cells and cellular regions by regulating key physiological processes, such as selectively interacting with enzymes or receptors, and modulating signaling pathways [2]. Therefore, accurate annotation of molecules is essential, and clear standards for molecular annotation have been adopted by the metabolomics community for bulk samples [3, 4, 5]. Yet, annotating and spatially localizing isomers within biological tissues remains a central challenge [6].

Mass spectrometry imaging (MSI) has emerged as a cornerstone for localizing molecules in situ by enabling highly multiplexed and label‐free spatial mapping of metabolites, lipids, and other biomolecules [7, 8, 9]. The capabilities of MSI have transformed investigations of tissue biochemistry, cellular heterogeneity, and pathological mechanisms [10, 11]. For example, MSI has revealed localized metabolic reprogramming as a key driver of cancer progression [12], spatially resolved metabolic interplay at the host–microbe interface [13], and drug distributions in pharmacological studies [14]. These spatially resolved molecular insights, unattainable through bulk analysis, have uncovered previously unrecognized chemical heterogeneity and critical molecular microenvironments that influence disease progression, metabolic regulation, and therapeutic insights.

Despite the advances, a key limitation of MSI is its inability to distinguish isomeric and isobaric species, even when augmented by high mass resolution or ion mobility [15]. Consequently, confidence in molecular annotation at isomeric level remains low in MSI workflows [6, 16]. By combining MSI with tandem mass spectrometry (MS^2^) in selected regions, using untargeted strategies such as data‐dependent and dataset‐dependent acquisition workflows, valuable structural information can be achieved [17, 18]. However, untargeted strategies do not fragment precursor ions consistently across the tissue and thereby the distribution of isomers remains hidden [19, 20].

In tandem mass spectrometry imaging (MS^2^I), each pixel contains consistent molecular fragmentation data, enabling visualization of product ion distributions, which facilitates structure‐resolved imaging [21, 22]. However, beyond the requirement of consistent precursor ion selection across pixels, current MS^2^I implementations are constrained by inherent trade‐offs among spatial resolution, throughput, and molecular coverage [21, 23, 24, 25, 26, 27]. To enable biological interpretation, it is essential that data acquisition and processing workflows provide confident molecular annotation and spatial distributions with isomeric specificity, this requires large‐scale, high‐throughput, structure‐resolved imaging that lies beyond the current state of the art.

Here, we present a scalable MS^2^I platform that advances MSI by integrating parallel image acquisition (PIA) with the computational framework spatial similarity network (SSN). PIA enables multiplexed parallel acquisition of high‐mass resolution untargeted MSI and scalable targeted MS^2^I data, structure‐resolved imaging of hundreds of molecular species with conserved pixel size and experimental time. Using SSN framework, molecular annotation is enhanced by exploiting the distribution of product ions and computing their spatial correlations to identify isomer annotations. We demonstrate our novel PIA SSN workflow to spatially map isomeric sterols and phospholipids in human and mouse brain, respectively. Overall, PIA and SSN together establish a pipeline for large‐scale, structure‐specific MSI, advancing spatial‐omics.

## 2 Results and Discussion

### 2.1 Scalable Structure‐Resolved Imaging via Parallel Image Acquisition

**FIGURE 1:**
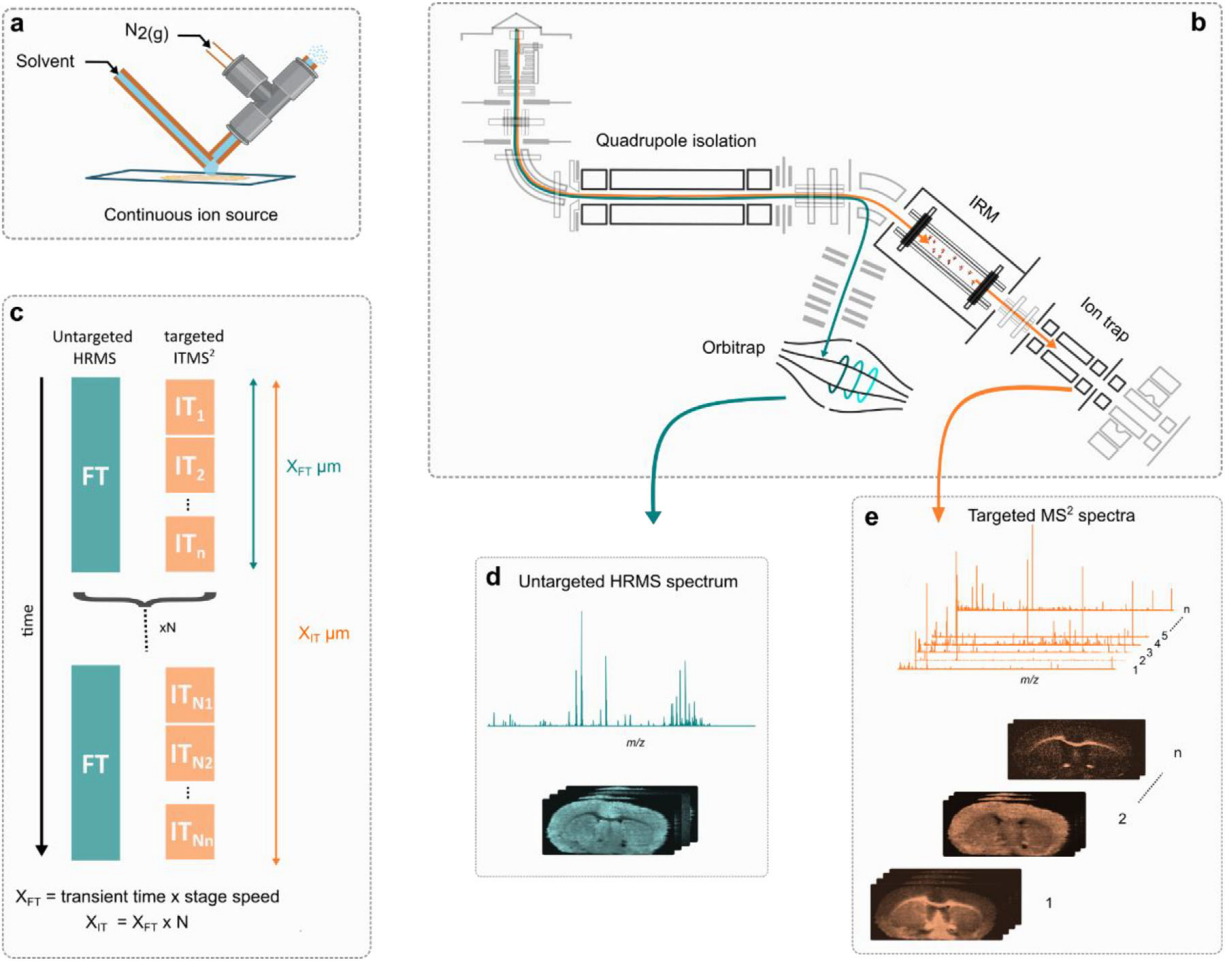
Schematic of Parallel Image Acquisition (PIA) enabling scalable MS^n^I. (a) PA nano‐DESI enables continuous sampling and ionization of molecules from tissue surfaces under ambient conditions, generating a high and continuous ion flux. (b) A tribrid mass spectrometer equipped with a quadrupole, ion trap, and orbitrap allows simultaneous acquisition of FTMS and ITMS^2^ data, enabling PIA. (c) Schematic of the PIA strategy: one high‐resolution FTMS scan is acquired in parallel with *n* targeted ITMS^2^ scans per pixel. Cycling through *N* inclusion lists extends precursor coverage, enabling scalable MS^2^ imaging without increasing acquisition time. (d) PIA produces untargeted, full‐scan, high‐mass resolution FTMS spectra with spatial information. (e) Simultaneously acquired targeted MS^2^ spectra and corresponding product ion images allow for structural and spatial resolution of molecular species at scale.

The ambient MSI technique pneumatically assisted nanospray desorption electrospray ionization (PA nano‐DESI) provides a continuous, high ion flux, offering exceptional sensitivity for MSI of low‐abundance metabolites, such as prostaglandins and steroids, which are generally difficult to ionize (Figure 1a) [28, 29, 30]. When coupled with a hybrid mass spectrometer, the high ion flux supports sequential multistage MS^n^ imaging (MS^2^I, MS^3^I, and MS^4^I), enabling isomer differentiation that is otherwise inaccessible [20, 23, 30, 31, 32]. Integrating PA nano‐DESI with a tribrid mass spectrometer, comprising a quadrupole mass filter, an Orbitrap (FT), and an ion trap (IT) mass analyzer, each operating independently, makes parallel data acquisition in the FT and IT possible [26]. The parallel acquisition strategy enables high mass resolution full‐scan FTMS while simultaneously performing serial fragmentation of targeted isolation windows in the IT at every pixel. In addition to maximizing the ion utilization, the additional data is acquired in parallel, so it does not extend acquisition time or compromise pixel size (Figure 1b,c).

The efficiency gain is especially striking in a typical FT scan (*m/Δm* = 500 000 at *m/z* 200, 1024 ms scan time, 5 ms ion accumulation time), where more than 99.5% of ions entering the spectrometer are otherwise unused. Parallel acquisition overcomes this inefficiency by utilizing the continuous ion current to acquire multiple ITMS^2^ spectra simultaneously within a single FT scan. In one experiment, 27 ITMS^2^ spectra can be acquired during a single high mass resolution FT scan while keeping identical spatial resolution for the FTMS and ITMS^2^ (Figures 1c–e and S1a). However, increasing the number of MS^2^ scans inevitably reduces the duty cycle for individual precursor ions, thereby reducing the signal‐to‐noise ratio and, consequently, limiting the achievable molecular coverage. To circumvent this limitation and expand total molecular coverage for MS^2^I, we loop through multiple inclusion lists (Figure 1c). For example, cycling through four inclusion lists, each targeting 27 narrow precursor isolation windows (0.7 Da), enables acquisition of MS^2^I data and product ion images from 108 precursor ion isolation windows in a single PA nano‐DESI MSI experiment (Table S1). As a trade‐off for looping multiple lists to gain increased structural information and visualizing thousands of product ion images in a single experiment, the pixel size in the x‐dimension for ITMS^2^ increases by a factor *N*, which corresponds to the number of inclusion lists, resulting in a lower spatial resolution compared to the FTMS (Figures 1c and S1b,c). Nevertheless, in nano‐DESI, the pixel size in the x‐dimension can be reduced by decreasing the stage speed and the pixel size in the y‐dimension by reducing the step size, through smaller capillary sizes or oversampling [33, 34, 35]. Here, we introduce this highly multiplexed workflow as parallel image acquisition (PIA) that enables the combination of non‐targeted full‐scan high mass resolution MSI in parallel with large‐scale targeted MS^2^I for precise spatial localization of product ions in a single, scalable experiment.

### 2.2 Decoding Complex MS^2^I Data With Spatial Similarity Networking (SSN)

**FIGURE 2:**
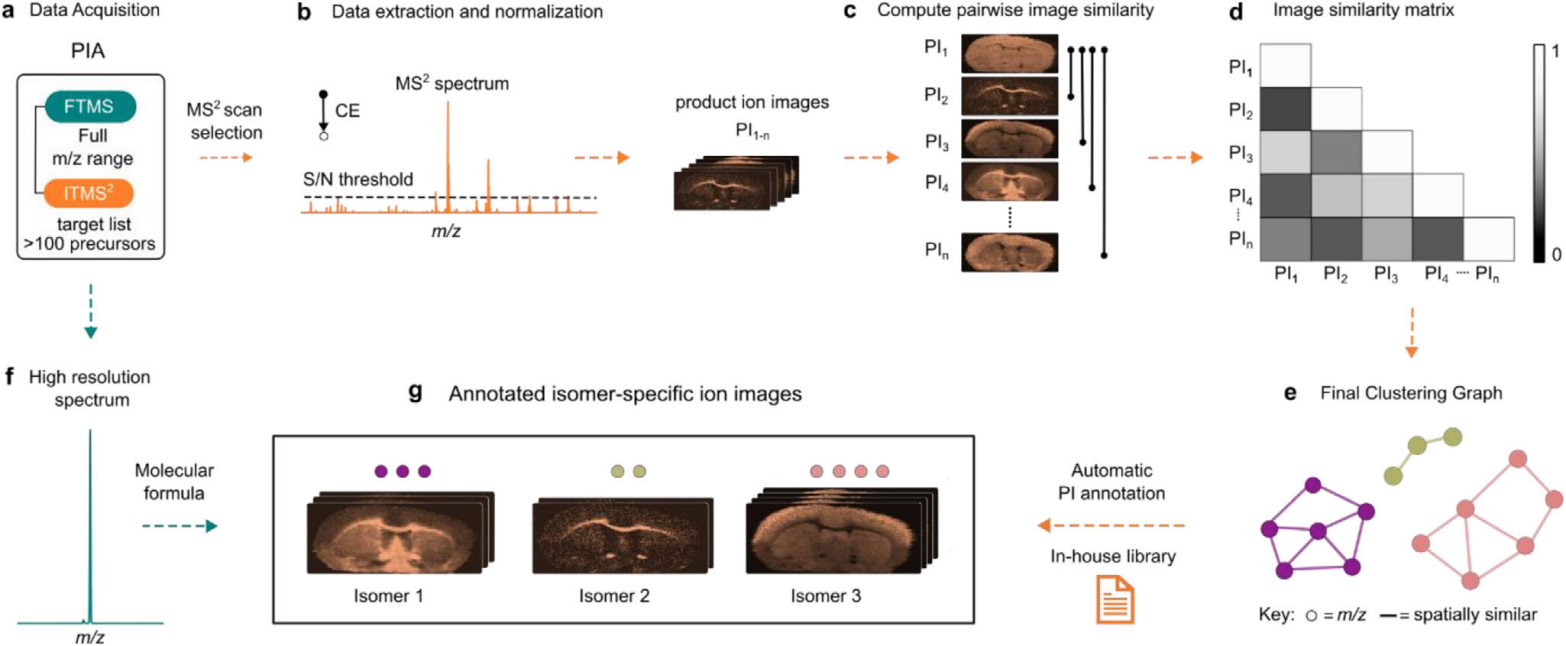
Schematic of spatial similarity networking (SSN) for deconvoluting complex MS^2^I data and enabling structure‐resolved annotation. (a) Full‐scan FTMS imaging is acquired across the entire *m/z* range and targeted MS^2^ spectra are acquired in parallel using narrow isolation windows (0.7 Da). (b) MS^2^ spectra from a selected isolation window are reconstructed into individual product ion images. (c) Pair‐wise spatial similarity is computed between product ion images using either SSE, MSE, or cosine similarity. (d) Similarity matrix for product ions in each precursor isolation window is generated. (e) Product ions are clustered using a connected components algorithm based on spatial similarity. Example output networks illustrate structurally distinct clusters arising from isomeric or isobaric precursors. (f) The molecular annotations are backed by high‐mass resolution data. (g) Each spatially coherent cluster is matched against a curated in‐house product ion library to automatically propose molecular annotations. SSN leverages spatial distributions as a third dimension of evidence, complementing accurate mass and fragmentation to enhance annotation confidence. PI: product ion.

The high multiplexity of each PIA experiment generates large, multidimensional datasets in which each pixel contains one full‐scan FTMS spectrum together with 27 targeted ITMS^2^ spectra from distinct isolation windows, encompassing multiple unique precursors (Figure 2a). Annotating molecules from such complex, multiplexed product ion spectra presents a formidable challenge. Existing software tools are designed for molecular annotations based on MS^1^ or isolated MS^2^ spectra from selected tissue regions, which renders them unsuitable for MS^2^I datasets that provide MS^2^ spectra at every pixel, yielding not only the product ions but also the corresponding spatial distributions [17, 36, 37, 38]. To address this gap, we have developed a spatial similarity networking (SSN) software tool which deconvolutes MS^2^I data by exploiting spatial correlation among product ion distributions. Specifically, the SSN leverages the fact that product ions derived from the same precursor exhibit identical spatial distributions, enabling clustering of product ions belonging to the same molecule for structural assignment. The resulting clusters do not overlap since one product ion will only belong to one cluster. This principle for structural assignment is analogous to the use of retention time in chromatographic separation; however, instead of retention time, the spatial distribution of product ions serves as an additional orthogonal dimension, alongside accurate mass and product ion spectra in the SSN.

The SSN framework works by comparing and scoring the spatial distribution between individual product ions and represents the results as a network. For each pixel, the SSN algorithm extracts MS^2^ spectra and computes a pairwise spatial similarity score between the product ion images using either cosine similarity, sum of squared error (SSE), or mean of squared error (MSE) metrics (Figure 2b–d, Supporting Information experimental section). While SSE and MSE account for absolute intensity, cosine similarity is independent of absolute intensity; therefore, it is particularly useful for comparing datasets acquired under different experimental conditions (Figure S2). From the similarity matrix, an undirected network is constructed in which nodes represent individual product ions and the edges denote spatial correlation (Figure 2e). Specifically, an unsupervised graph‐based clustering, that uses a connected components algorithm, identifies groups of product ions that share the same distribution, efficiently removing noise and randomly generated product ions. Importantly, this is achieved without prior knowledge of precursor identity, number, or fragmentation pathways. By treating each product ion image as an independent variable, the SSN enables structural annotation to be derived directly from spatially coherent ion clusters. In doing so, SSN introduces spatial distributions as a third orthogonal dimension, alongside accurate mass and fragmentation patterns, to facilitate unambiguous molecular annotation, including isobars and isomers. A detailed description of the SSN workflow is provided in the experimental section of the Supporting Information with a step‐by‐step guideline.

Note that the SSN is compatible with any MS^2^I imaging workflow, irrespective of the ion source or tandem mass spectrometer, provided the same precursor ion is targeted using a narrow precursor isolation window throughout the experiment to generate product ion images. This compatibility arises because SSN operates on a single MS^2^ scan filter at a time and calculates similarity scores based on product ion distributions. Beyond MS^2^I, SSN also enables non‐targeted exploration of full‐scan MSI data where similarly distributed molecules are clustered based on spatial similarity (Figure S3). Furthermore, for highly heterogeneous tissue, improved robustness is achieved by a coherent SSN workflow. This workflow utilizes patch‐based image decomposition, which emphasizes regional spatial coherence while suppressing localized noise, thereby improving clustering performance (Figure S4).

The SSN tool is integrated into our freely available *ion‐to‐image* (i2i) software package and support the common .mzML data format, ensuring accessibility across imaging platforms (Figure S5) [39]. Within i2i, automatic structural annotation is possible. By uploading a library that is paired with product ions, the spatially clustered networks of product ions can be matched and annotated (Tables S2–S4). When SSN is combined with PIA, annotation can be further validated by extracting the product ions of individual clusters and correlating these with the molecular formula achieved from the simultaneously acquired FTMS data (Figures 2f,g, and S5). Overall, the SSN tool yields a robust, multidimensional framework for confident molecular annotation by utilizing the spatial distributions of product ions.

### 2.3 Isomer‐ and Isobar‐Resolved Molecular Imaging and Annotation

**FIGURE 3:**
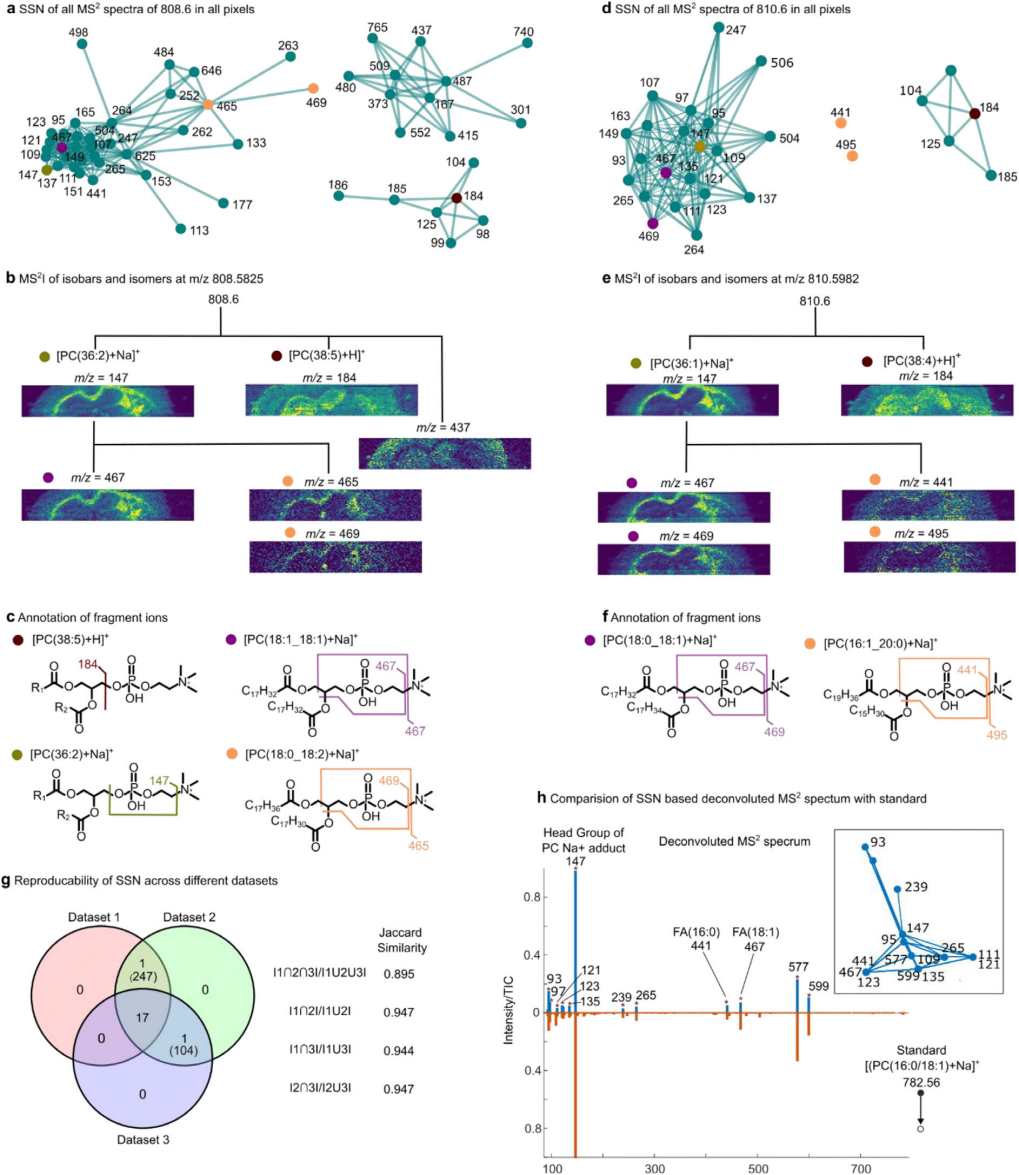
Combined PIA and SSN workflow reveal molecular distributions hidden from conventional MSI. (a) SSN constructed from product‐ion images obtained by fragmentation of *m/z* 808.6. Three distinct clusters of product ions with unique spatial distributions are observed, indicating the presence of multiple co‐isolated precursors within a single isolation window. (b) Corresponding MS^2^ ion images for representative product ions from each SSN cluster, illustrating distinct molecular distributions across the tissue. (c) Higher‐energy collisional dissociation of phosphatidylcholines (PCs) produces diagnostic head‐group and acyl‐chain fragments, revealing the distribution of co‐isolated isobars such as [PC(38:5)+H]^+^ and [PC(36:2)+Na]^+^, as well as isomeric species including PC(18:1_18:1) and PC(18:0_18:2). (d‐f) Fragmentation of *m/z* = 810.6 revealed the distribution of two isobars [PC(38:4)+H]^+^ and [PC(36:3)+Na]^+^ and two isomers PC(18:0_18:1) and PC(16:1_20:0). SSN is reproducible across different datasets. (g) Venn diagram summarizing the overlap of product ions from SSN‐derived clusters generated from MS^2^ fragmentation of *m/z* 782.56 across three independent MS^2^I datasets. A total of 19 unique product ions were detected, of which 17 were shared across all datasets. Pairwise Jaccard similarity was used to quantify SSN reproducibility. (h) Comparison of an SSN‐resolved, deconvoluted MS^2^ spectrum with a lipid standard. The SSN‐derived product‐ion cluster assigned as [PC(16:0_18:1)+Na]^+^ (PC(34:1)+Na^+^) is shown in the insert above the reconstructed MS^2^ spectrum (top), with the reflected MS^2^ spectrum of the corresponding standard shown below. All fragment ions recovered by SSN are above 1% of the base peak intensity in the standard MS^2^ spectrum. For experimental details, see Dataset 1 in Table S2, for SSN details, see Table S12. Pixel sizes of 20 × 50 µm for FTMS and 80×50 µm for ITMS^2^. Note the color code in (a) and (d) is to help identify the ion images and fragmentation shown in (b and c) and (e and f), respectively.

Phospholipid isomers and isobars individually regulate cell membrane properties, signaling, and energy storage, making accurate structural annotation crucial for understanding their biological role [40, 41, 42]. Variations in phospholipid head group, chain length, saturation, and fatty acyl positions contribute to numerous unique lipid species with near‐identical *m/z* values that cannot be resolved with conventional MSI. However, with the PIA and SSN workflow, we report over 100 annotated phospholipid isomers and isobars in mouse brain tissue in a single experiment. Specifically, the PIA experiment included one FT scan (at 500 000 resolving power at *m/z* 200) and 108 parallel ITMS^2^ scans (4 loops x 27 isolation windows/pixel; ±0.35 Da; HCD). This generated multiple co‐isolated precursors in each isolation window and over 800 000 MS^2^ spectra per imaging experiment with pixel sizes of 20 × 50 µm for FTMS and 80 × 50 µm for ITMS^2^ (Figures 3 and S6).

Out of the 108 isolation windows, 34 belonged to phospholipid species. Within the isolation window centered at *m/z* 808.6, the SSN revealed three discrete product ion clusters with distinct spatial distributions, indicating the presence of at least three different precursors in this *m/z* window (Figures 3a,b and S7). To identify the precursor ions underlying each cluster, the product ions were first automatically annotated using our in‐house generated phospholipid library, which contains predicted product ions from rule‐based fragmentation patterns (Tables S2–S4) [43]. Next, molecular formulas were assigned from the accurate mass measured by FTMS. Finally, the annotated product ions were combined with the molecular formulas to determine the phospholipid structures down to fatty acid acyl chain composition (Figures 3c, S8, and S9). This workflow enabled confident assignment of the three SSN clusters as the three isobars: [PC(36:2)+Na]^+^ (*m/z* 808.5832), [PC(38:5)+H]^+^ (*m/z* 808.5856), and [PC(O‐36:3)+K]^+^ (*m/z* 808.5622). Further inspection of the SSN revealed that [PC(36:2)+Na]^+^ comprised two structural isomers, [PC(18:1_18:1)+Na]^+^ (*m/z* 467) and [PC(18:0_18:2)+Na]^+^ (*m/z* 465 and 469). While both isomers were detected in the corpus callosum, [PC(18:0_18:2)+Na]^+^ also extended to the lateral ventricles, reflected by the higher‐intensity central feature in the corresponding product ion images. Crucially, without MS^2^I and SSN, neither the isobars [PC(36:2)+Na]^+^ and [PC(38:5)+H]^+^ nor the isomers within [PC(36:2)+Na]^+^ could be distinguished in the FTMS spectrum, even at the high mass resolving power.

In another example, within the isolation window at *m/z* 810.6, the SSN revealed two co‐isolated isobars [PC(38:4)+H]^+^ and [PC(36:1)+Na]^+^, which cannot be distinguished by accurate mass alone (Figure 3d). Within the [PC(36:1)+Na]^+^ cluster, SSN further resolved the two isomers [PC(18:0_18:1)+Na]^+^ and [PC(16:1_20:0)+Na]^+^ based on their distinct spatial distributions and the markedly lower signal intensity of [PC(16:1_20:0)+Na]^+^ (Figures 3d–f and S10–S11) Similarly, SSN enables annotation of isomers from other phospholipid classes. For example, co‐isolated phosphatidylethanolamine (PE) isomers were resolved and annotated based on spatially correlated product ions using SSN (Figure S12 and Table S6). Notably, product ions with low S/N may not be consistently detected across pixels, increasing variability in spatial similarity scores and causing these ions to remain as singlets at high similarity thresholds (e.g., *m/z* 441 and *m/z* 495 in Figure 3d). Lowering the similarity threshold can promote clustering of such ions, although this also increases the risk of incorporating noise or unrelated signals. Importantly, SSN does not discard these ions; they remain available for annotation using rule‐based fragmentation patterns.

The robustness and reproducibility of SSN clustering were validated across multiple datasets, tissue sections, and inclusion lists (Tables S6 and S7). To quantitatively assess reproducibility across biological replicates, we applied the workflow to the same isolation window from three independent mouse brain datasets. Using a consistent clustering threshold, the clusters from the three different datasets all included the same 17 product ions and when comparing the clusters using the Jaccard similarity index, a value of 0.895 was returned (Figures 3g and S2). This high similarity confirms that SSN reliably groups product ions based on spatial distributions, even across different tissue sections. Beyond cross‐dataset reproducibility, the SSN‐based deconvoluted MS^2^ spectrum was validated by comparison to an authentic endogenous standard. For the [PC(16:0_18:1)+Na]^+^ precursor, the SSN‐reconstructed MS^2^ spectrum showed complete agreement with the standard spectrum for all fragment ions above 1% of the base peak intensity (Figures 3h and S13). This result confirms that spatial similarity among product ions provides a robust strategy for deconvoluting and annotating chimeric MS^2^ spectra in MSI. Collectively, these examples demonstrate that SSN resolves chimeric MS^2^ data by separating unrelated isobars and background ions. In addition, MS^2^I and spatial clustering by SSN can reveal distinct spatial distributions of isomers and isobars that remain unresolved even at high mass resolving power.

### 2.4 Classification of Confidence Levels in MSI Annotation

**FIGURE 4:**
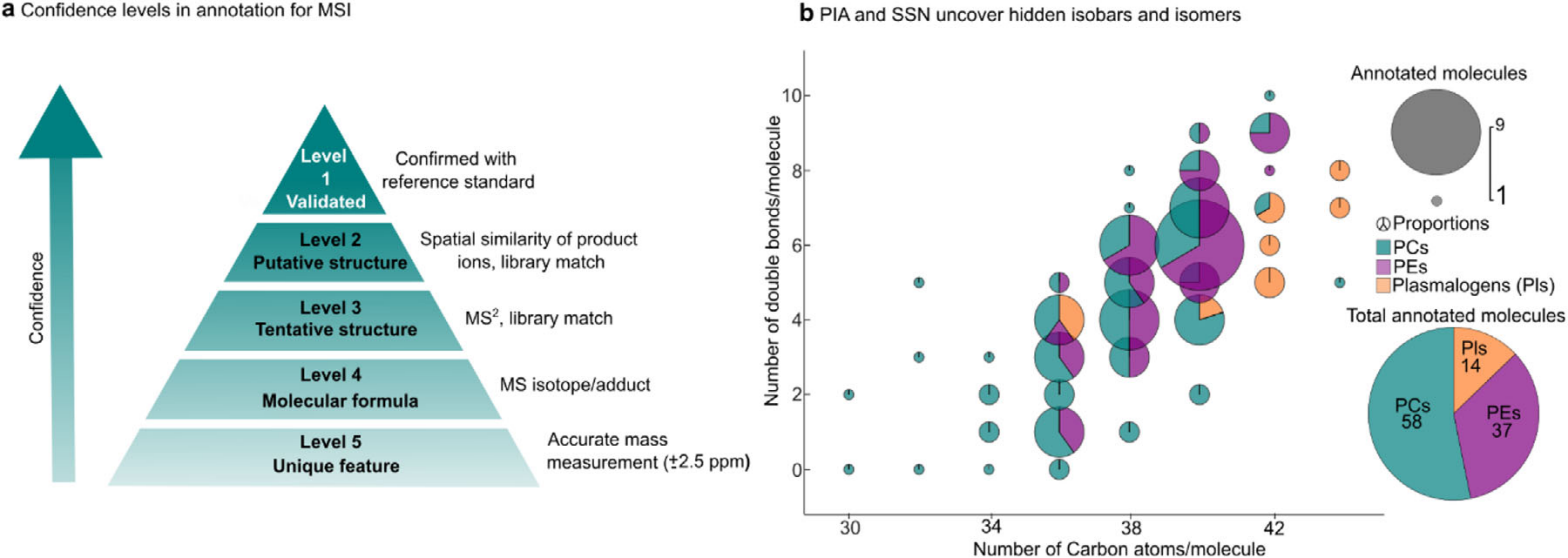
Annotation in MSI. (a) Proposed confidence levels for MSI annotation. Spatial similarity of product ions across all pixels is required at Level 2 and can be demonstrated using SSN. (b) The bubble pie chart summarizes PIA SSN‐based results from 34 precursor isolation windows, identifying 134 lipid species, including phosphatidylcholines (PCs), phosphatidylethanolamines (PEs), and plasmalogens (Pls), annotated at Level 2 confidence. Out of these, 109 were unique phospholipid structures corresponding to 58 PCs, 37 PEs, and 14 PE plasmalogens/alkyl ethers that could not be uniquely resolved by MS^1^ imaging alone, highlighting the power of SSN for resolving isobaric and isomeric complexity in situ.

High‐throughput molecular annotation is a critical component of chemical analysis, and most research communities rely on standardized guidelines with defined confidence levels [5]. However, for the MSI field, annotation guidelines are still lacking despite substantial technological advances. Therefore, we propose a new guideline for classifying confidence levels in molecular annotation in MSI (Figure 4a, Table S8). This system ranges from Level 5, where annotation is based solely on accurate mass, to Level 1, where confirmation against a reference standard and orthogonal validation are required, which is generally impractical at the pixel level for MSI. Level 3 annotations are based on accurate mass together with MS^2^ data, typically acquired from a few pixels on the tissue. While valid, this approach provides only limited confirmation on the distribution across the tissue section and does not capture the full spatial distribution of product ions. Level 2 annotations, in contrast, require fragmentation data from all pixels over the tissue and, critically, the clustering of product ions to demonstrate spatial similarity above a defined threshold. This ensures that product ions are consistently linked to their precursors, enhancing reproducibility and structural specificity. In this guide, SSN‐based clustering of product ions and MS^2^ library matches of clusters is classified as confidence Level 2.

Using the SSN across the 34 phospholipid targeted isolation windows within the PIA data set, we achieved Level 2 annotation of 134 distinct isomeric and isobaric lipid species, including 70 phosphatidylcholines (PCs), 44 phosphatidylethanolamines (PEs), and 20 PE‐plasmalogens (Figure 4b). The same species were detected and annotated across several PIA datasets (Table S6). It is important to note that 75 (55%) of these species were not resolvable by FTMS imaging alone, despite the high mass resolution (*m/Δm* = 500 000 at *m/z* 200). In addition to revealing superimposed isobaric and isomeric features, the spatial–spectral concordance significantly reduces the risk of false annotations, offering a robust strategy for interpreting MSI datasets and advancing molecular imaging toward more confident structural assignment. Overall, the PIA and SSN workflow provides a multidimensional framework for confident structural annotation, with a solid foundation essential for biological interpretation.

### 2.5 Annotating and Visualizing Sterol Lipids in Human Multiple Sclerosis Brain Tissue

**FIGURE 5:**
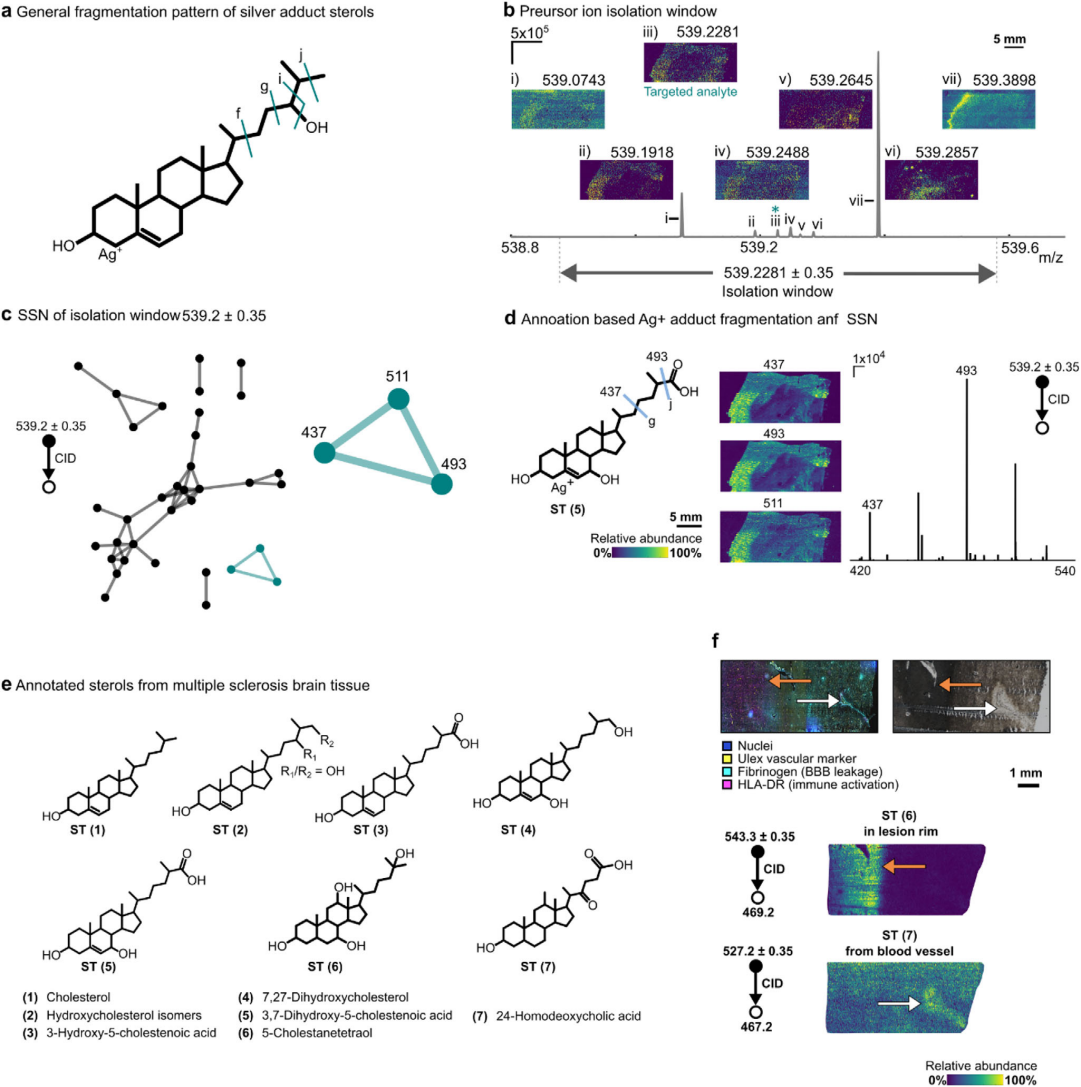
Detection and annotation of sterol lipids in human multiple sclerosis brain tissue. (a) Sterols, ionized as silver adducts, exhibit consistent fragmentation in their side chains as illustrated for 24‐hydroxycholesterol. (b) An FTMS spectrum detailing the narrow isolation window (0.7 Da) of ions selected for ITMS^2^ with corresponding high‐mass resolution precursor ion images. (c) SSN clusters all product ion images based on spatial similarity. The network highlighted in green corresponded to the side chain fragmentation of a sterol lipid fragmented as silver adduct. (d) Side chain fragmentation matches with 3, 7‐dichydroxycholestenoic acid, and the three clustered product ions show very similar distributions, indicating they arise from the same molecule. (e) The structure of cholesterol and six annotated oxysterol species from human multiple sclerosis brain tissue. (f) A multimodal study combining PIA, SSN, and histological staining reveals infiltration of homodeoxycholic acid to a white matter lesion through a BBB leakage site. Representative image of UEA‐I, Fibrinogen, and HLA‐DR immunoreactivity and optical image with corresponding images of diagnostic product ions for cholestanetetraol (ST 6) and 24‐homodeoxycholic acid (ST 7), white arrow: BBB leakage site. BBB: blood–brain barrier, CID: collision‐induced dissociation. For experimental details see Dataset 5 and 7 in Table S7, for SSN details see Table S11.

Sterol lipids regulate essential biological processes and exist in many isomeric forms due to variations in unsaturation and hydroxylation [42, 43, 44]. Cholesterol, a major sterol lipid linked to demyelination, which is the hallmark of multiple sclerosis, shows altered levels in body fluids of people with multiple sclerosis (PwMS), along with other sterol lipids [44, 45]. However, the spatial distribution of distinct sterol lipids in PwMS brain tissue, which can provide insights into cholesterol clearance and disease‐related pathways, remains unknown due to their low abundance, poor ionization efficiency, and extensive isomerization [46, 47, 48]. Here, we chemically mapped human multiple sclerosis brain tissue using silver‐doped PA nano‐DESI and PIA and targeting sterol lipids (Figures 5a,b and S14–S15) [29]. The chemical complexity of this tissue is high; for example, within a selected isolation window of *m/z* 539.2 ± 0.35 Da, dihydroxycholestenoic acid, the FTMS spectrum showed seven co‐isolated precursor ions. Each precursor ion has a distinct spatial distribution over the tissue, and each contributes to the MS^2^ spectrum (Figure 5b). Despite this complexity, SSN effectively deconvoluted the MS^2^ spectra and clustered the product ions into six distinct groups (Figure 5c). One cluster, comprised of three product ions with highly similar spatial distributions, was annotated as dihydroxycholestenoic acid (ST5) based on established diagnostic product ions (Figure 5d and Table S9) [42]. In total, we successfully mapped the distribution of cholesterol and six sterol lipids within human brain tissue using the PIA and SSN workflow (Figures 5e and S16–S18). For example, the accumulation of 5‐cholestenetetraol (ST6) was observed in a highly immunoreactive lesion rim (Figure 5f). Moreover, the localization of the bile acid 24‐homodeoxycholic acid (ST7) from a blood vessel was revealed indicating its higher levels in the blood (Figure 5f) [49]. Since ST6 and ST7 sterols are not biosynthesized within the brain, our results support the occurrence of BBB disruption in multiple sclerosis pathology [50]. These findings demonstrate that the combination of PIA and SSN resolves low‐abundance, co‐isolated sterols in complex human tissue, enabling MSI of isomers and isobars beyond current state‐of‐the‐art methods.

**FIGURE 6:**
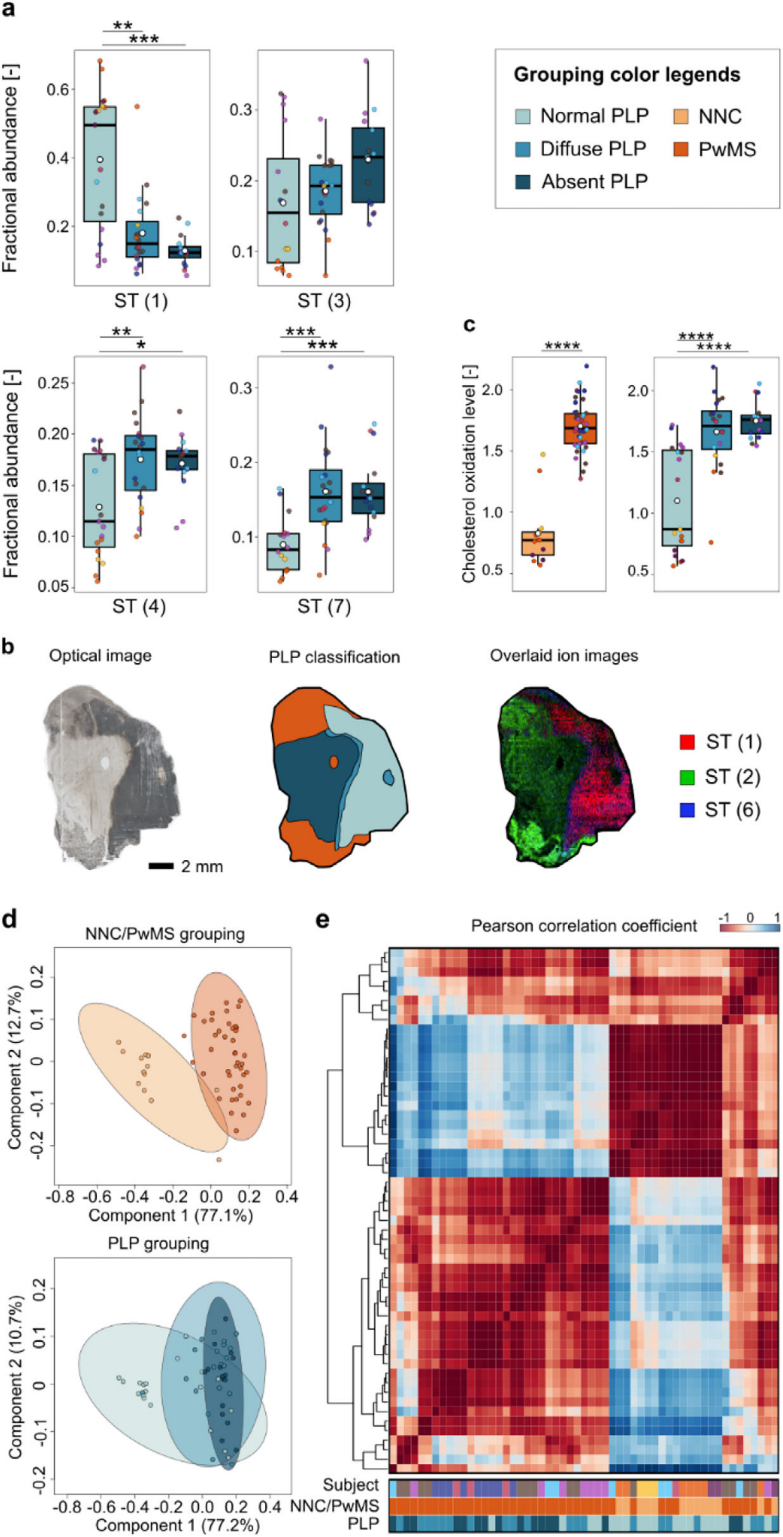
The landscape of cholesterol and cholesterol oxidation products is altered in multiple sclerosis. (a) Boxplots represent the fractional abundance of significantly decreased cholesterol (ST 1) and increased oxysterol (ST 3, ST 4, ST 7) species. (b) Overlaid ion images of ST1, ST2, and ST6 in an example PwMS tissue section show the opposite spatial distribution of cholesterol and oxidized sterols. (c) Levels of sterol oxidation significantly increases in diffuse and absent PLP category compared to normal PLP and is higher in PwMS compared to NNCs. For calculations see Methods section. Point colors indicate individual donors as per Table S1, black line denotes the median and the white point the mean. (d) PLS‐DA analysis of sample grouping shows considerable differences according donor type (NNC vs PwMS) and slight separation of absent PLP from normal and diffuse PLP groups based on oxysterol profiling of ROIs. (e) Pearson correlation heatmap of ROIs reinforces the PLS observations by showing primary clustering based on donor type and secondary clustering based on PLP grouping. PLP, proteolipidprotein staining of myelin; NNC, non‐neurological control; PwMS, people with multiple sclerosis. (*:*p*<0.05, **:*p*<0.01, ***:*p*<0.001, ****:*p*<0.0001). For experimental details see Dataset 8 in Table S7.

The sterol lipids ST2‐ST5 are formed by oxidation of cholesterol and may be directly linked to lesion formation and disease progression in multiple sclerosis (Figure S18) [44]. By combining silver‐doped PA nano‐DESI MSI, PIA, and immunohistochemical techniques to determine and annotate regions of interest (ROI), we found a significant 2‐fold depletion of cholesterol that correlates with demyelinated regions (Figures 6a and S19). Additionally, the ST4 and ST7 were significantly increased (∼2‐fold) in regions exhibiting diffuse and absent PLP compared to areas with normal PLP (Figure 6a). Furthermore, an increasing trend was observed for ST2 and ST3, which are indicated as being neurotoxic (Figures 6a and S20) [51]. Looking at the overall oxysterol landscape, cholesterol was found to be the dominant sterol in NNC tissues and normal PLP regions, while in PwMS and absent PLP areas, hydroxycholesterol was most abundant (Figure S20). For example, the spatial distribution of ST1, ST2, ST6 clearly matches the PLP classification of the tissue section (Figure 6b). Together with the relative increase of other oxidized sterols, this shows increased cholesterol oxidation levels in diffuse and absent PLP areas compared to normal PLP (Figure 6c). The increased oxidation levels are even more pronounced when comparing tissue from NNC and PwMS (Figure 6c). Moreover, a considerable difference in total oxysterol profiles was observed when grouping ROIs based on NNC or PwMS (Figures 6d,e, and S20). This indicates that the decreased cholesterol level and elevated cholesterol oxidation are a combination of myelin degradation and other effects, including local inflammatory and immune response. Overall, using our multimodal platform, we reveal an interchange between cholesterol and oxidized cholesterol species during demyelination in multiple sclerosis and elevated cholesterol oxidation in brain tissue from PwMS, underscoring the value of enabling spatially resolved, isomer‐specific imaging.

## 3 Conclusion and Future Perspectives

We present a novel approach for structurally resolved MSI by combining PIA and SSN in a powerful platform for high‐precision spatial omics. This facilitates both extensive molecular coverage from MS^2^I and detailed molecular structure analysis based on spatial distribution, with the SSN seamlessly integrated into our i2i software and compatible with any MSI or MS^2^I data. We show the distribution and detailed molecular structure characterization of isomeric and isobaric phospholipids in mouse brain tissue in addition to the much less abundant oxysterols in human brain tissue. Additionally, we suggest new annotation levels for strengthening reported annotation by MSI, increasing validity for the entire MSI community. In the context of multiple sclerosis, we found implications of cholesterol oxidation in multiple sclerosis lesions. While our applications primarily highlight metabolomics, the PIA and SSN strategies are versatile and applicable to proteomics, glycomics, and pharmacokinetics. Moreover, coupling to higher spatial or tandem mass resolution instrumentation [52], or reactive chemistry workflows [19, 53], could further deepen structural characterization. Ultimately, both PIA and SSN provide new approaches to integrating large‐scale, information‐rich molecular distributions with enhanced structural characterization, setting a new benchmark in spatial‐omics.

## Author Contributions

V.V.S and G.T. contributed equally and share first authorship of the manuscript.

## Conflicts of Interest

The authors declare no conflicts of interest.

## Code Availability Statement

The source code and the compiled version of the software are available at https://github.com/LanekoffLab.

## Supporting information




**Supporting File 1**: anie71994‐sup‐0001‐SuppMat.pdf.

**Supporting File 2**: anie71994‐sup‐0002‐SuppMat.xlsx.

## Data Availability

Data presented in the manuscript are available from the corresponding author upon reasonable request.
