# Supplementary material for: Multiplexed Tandem Mass Spectrometry Imaging Enables Large‐Scale Isomer Mapping and Annotation in Tissues
Source: Angew Chem Int Ed Engl. 2026 Mar 30;65(20):e22119. doi: 10.1002/anie.202522119 (PMC13159427; doi:10.1002/anie.202522119)
Supplement: Supplementary file 1 — Supporting File 1: anie71994‐sup‐0001‐SuppMat.pdf. [file ANIE-65-e22119-s001.pdf]

# Supplementary information

## Multiplexed tandem mass spectrometry imaging enables large-scale isomer mapping and annotation in tissues


Varun V. Sharma[1]*, Gabor Toth[1,2]*, Robert Martinis[1], Cathrin E. Hansen[3,4,5], Gijs Kooij[3,4,5,6], Ingela Lanekoff[1,2#]

[1] Department of Chemistry for Life Sciences, Uppsala University, Uppsala, Sweden

[2]Center of Excellence for the Chemical Mechanisms of Life, Uppsala University, Sweden

[3] Amsterdam UMC, Location VU Medical Center, Department of Molecular Cell Biology and Immunology, De Boelelaan 1117, Amsterdam, The Netherlands

[4] Amsterdam Neuroscience, Amsterdam UMC, Amsterdam, The Netherlands

[5] MS Center Amsterdam, Amsterdam UMC, Location VU Medical Center, Amsterdam, The Netherlands

[6]Amsterdam Institute for Immunology and Infectious Diseases, Amsterdam UMC, Amsterdam, The Netherlands

* Both authors contributed equally to this work

[#] Corresponding author

**Corresponding author:**

**Prof. Ingela Lanekoff**

Ingela.Lanekoff@kemi.uu.se

Dept. of Chemistry for Life Sciences (576)

Uppsala University

751 23 Uppsala

Sweden

# Table of Contents

Ethical Approvals ........ 3

Experimental Section ........ 3

Step-by-step guideline for data acquisition and analysis ........ 7

List of supplementary tables ........ 8

Supplementary figures ........ 9

Figure S1 Parallel image acquisition (PIA) enables multiplexed $MS^2$ imaging without compromising acquisition time ........ 9

Figure S2 Comparison of fragment-ion clusters across three $MS^2$I datasets. ........ 10

Figure S3 The SSN tool enables non-targeted data exploration of $MS^1$I data ........ 11

Figure S4 Schematics of patch-wise coherent spatial similarity networking (coherent SSN) ........ 12

Figure S5 Graphical user interface of the new SSN module in i2i ........ 13

Figure S6 SSN of 108 targeted mass channels show a great versatility of spatial distributions ........ 14

Figure S7 Product ion network clusters and corresponding images from fragmentation of the isolation window centered at 808.60 ± 0.35 Da ........ 15

Figure S8 SSN works as a separation dimension unique to MSI and deconvolutes product ion mass spectra ........ 16

Figure S9 Fragmentation patterns of phosphotidylcholine (PCs) and phosphotidylethanolamine (PEs) as sodiated adducts in HCD ........ 17

Figure S10 Product ion image networks from PIA SSN ........ 18

Figure S11 SSN deconvolutes product ion mass spectra and facilitates annotation of isobars and isomers ........ 19

Figure S12 Annotation of phosphatidylethanolamine species $[PE(40:6)+Na]^+$ by SSN ........ 20

Figure S13 Chimeric MS2 spectra obtained from fragmentation of m/z 782.56. ........ 20

Figure S14 Fragmentation of argenated adducts of hydroxycholesterols ........ 21

Figure S15 The isolation window contains multiple precursor ions ........ 22

Figure S16 The SSN deconvolutes complex $ITMS^2$ spectra produced by the fragmentation of co-isolated precursor ions ........ 23

Figure S17 Detected oxysterol species from human multiple sclerosis tissue by sequential $FTMS^2$I ........ 24

Figure S18 Metabolic pathway for biosynthesis of bio acids from cholesterol ........ 25

Figure S19 Multimodal imaging of cholesterol and oxidized cholesterol products in human white matter brain tissue sections ........ 26

Figure S20 Differentiation and clustering of brain tissue regions based on their oxysterol profiles ........ 27

References ........ 28

# Ethical Approvals

**Human samples for Multiple Sclerosis study**

All human donors or their next of kin provided fully informed consent for autopsy and for the use of material for research at the Netherlands Brain Bank, under ethical approval from the Medical Ethics Committee of the Free University Medical Center in Amsterdam (2009/148), project number 1,127. Donor details are shown in Table S10.

**Mouse brain samples**

Mouse brain samples were purchased from Creative Biolabs (Shirley, NY, USA). Creative Biolabs conducts all experiments in accordance with the Institutional Animal Care and Use Committee (IACUC) requirements. The IACUC number for the project is GP02-010-2021v1.0. Note that no experiments were performed on live animals by the authors.

# Experimental Section

## Sample selection and preparation

Mouse brain (8–10 weeks old male C57BL/6) was purchased from Creative Biolabs (Shirley, NY, USA) and cut to a thickness of 10 µm using a cryo-microtome (Leica Microsystems, Wetzlar, Germany). The sections were thaw-mounted onto regular microscope glass slides and stored at -80 °C prior to analysis.

Freshly frozen post-mortem human brain samples were obtained from the Netherlands Brain Bank from 5 clinically diagnosed MS patients (mean age at death: 65 years) and 3 non-neurological controls (mean age at death: 66 years, Table S10). Tissue blocks were cut into 10 µm sections and stored at -80 °C until further use. Samples were gently thawed at room temperature under constant airflow before analysis. Mouse brain sections were analyzed without any sample preparation, human multiple sclerosis tissue sections were dipped six times in deionized water (Milli-Q, 18.2 MΩ) for one minute each to remove excess salts. For MSI, the samples were placed on an XYZ linear motor stage (Zaber Technologies Inc., Vancouver, BC), controlled via a custom-designed LABVIEW program.[1]

**Parallel image acquisition**

Mass spectrometry was performed in positive ion mode on an Orbitrap IQ-X (Thermo Fisher Scientific, San Jose, CA, USA) using high-resolution Orbitrap (FTMS) $MS^1$ and ion trap (ITMS) $MS^2$ modes, or $FTMS^2$ for accurate mass confirmation. For PIA, ions were first selected for a full FTMS scan and subsequently for several parallel $MS^2$ scans in the ion trap during the FTMS transition time.[2] The PIA methodology was tested across five different experimental conditions (varying extraction solvents and targeted analytes) to demonstrate its versatility. Detailed mass spectrometry acquisition parameters for each experiment are provided in Table S7.

## PA nano-DESI MSI setup and data extraction

The PA nano-DESI was set up as previously described,[3] using 150/50 µm o.d./i.d. fused silica capillaries (Genetek, Sweden). Briefly, the primary capillary, connected to a syringe containing the nano-DESI solvent, was coupled to a pneumatically assisted secondary capillary positioned at ~90 degrees. The extraction solvent was delivered through the primary capillary via a syringe pump (Legato 180, KD Scientific, Holliston, USA), with the ESI voltage applied directly to the syringe needle. The probe and

sample positioning were monitored using long-working-distance digital optical microscopes (Dino-Lite, USA). For all experiments, the internal Detailed PA nano-DESI parameters are provided in Table S7.

Figures showing data and metadata from the experiments were exported from FreeStyle 1.8 SP2, v1.8.63.0. Ion images were generated using our in-house software tool, i2i,[4] from .mzML data converted via MSConvertGUI (ProteoWizard, v3.0.22285) from .RAW files. The use of .mzML format facilitates a vendor-neutral use of the software. Ion images of the $MS^2$ data were created by imputing the scans on a master grid generated from the FTMS scans, where the specific $MS^2$ spectra were given the same coordinates as the closest FTMS master scan.

## Spatial similarity networking

Spatial similarity networking (SSN) is a custom software tool developed to deconvolve $MS^2$I datasets and is freely available as part of our i2i software.[4] SSN accepts $MS^2$I datasets as input and outputs both deconvoluted product ion networks, their corresponding spectra, and product ion images. In this study, SSN was applied to all datasets, including imaging of the mouse brain and human multiple sclerosis brain tissue. Deconvoluted networks enabled downstream structural annotation. Specific parameter sets used for generating the SSN plot are described in Table S11.

The SSN is computed based on the intensities of all identified *m/z* values in one scan filter over all pixels of the ion image. First, the user selects the scan filter, scan type, and search parameters. The parameters include (i) minimum and maximum intensities. (ii) selection of spatial similarity metric (cosine similarity, sum of squared error (SSE), or mean squared error (MSE)) iii) the similarity threshold used to define spatially correlated product ions. (iv) the mass accuracy appropriate for the dataset. Note that the GUI provides suggested pre-set parameters for ppm tolerance and min and max intensities; these may need to be altered based on the experiment.

When the user clicks the run button, a non-targeted search, based on the user-defined parameters, is conducted to identify *m/z* values that will be considered for the SSN. The algorithm for the search is identical to the algorithm in the non-targeted tab of i2i.[4] Following, one of the *m/z* values is selected as an *anchor ion image,* and the distance metric is calculated for all identified *m/z* values against the anchor ion image. To normalize the response of different ions, all compared *m/z* values are first scaled to the 99$^{th}$ percentile of intensity within their respective ion image. This allows for comparison across *m/z* values despite differences in absolute intensities. Subsequently, the anchor ion image is exchanged to ensure that all ion images of identified *m/z* values are compared with each other.

The equations used to calculate the SSN based on sum of squared error (SSE), cosine distance, and mean of squared error (MSE) are shown in Eqs. 1, 2, and 3, respectively. The x and y are intensities of the respective *m/z* in a given pixel, the $x_{anchor}$ is the matrix of the anchor ion image and $y_i$ is the matrix of the ion image for the $i^{th}$ ion. The N is the number of pixels in one image/patch being compared.

$$SSE = \sum |x_{anchor} - y_i|^2 \qquad \textit{Eq. 1}$$

$$Cosine\ distance(x_{anchor}, y_i) = 1 - \frac{x_{anchor} \cdot y_i}{\|x_{anchor}\| \|y_i\|} \qquad \textit{Eq. 2}$$

$$MSE = \frac{1}{N} \sum_{i=1}^{N} (x_i - y_i)^2 \qquad \textit{Eq. 3}$$

The computational process creates a lower triangular correlation matrix with values corresponding to the selected similarity index (SI). The use of a lower triangular matrix saves memory usage and computation time during the search process. The product ion images are grouped based on the user-selected percentile of the similarity index values to be included. For example, by selecting 0.1, only 10% of the lowest SI values (i.e. the SI values with the highest correlation) are used to generate a filtered correlation matrix. The error limit has to be optimized for each experiment with respect to the complexity of the given scan event. A graph in MATLAB is then created with the filtered correlation matrix, with each group in the correlation matrix corresponding to a connected component of the graph. Following, the function conncomp is used to extract the spatially correlated *m/z* values of the groups. The results are shown as clusters of *m/z* values with similar distributions, termed the SSN.

One cluster of the SSN includes all product ion images that have the same distribution based on the set parameters. Thus, one product ion will only appear in one cluster. To interrogate the SSN, the user can select groups and their *m/z* values in the list box next to the displayed SSN. Alternatively, by opening the SSN as a plotted figure, the user can hover over the displayed SSN to view information on each node in real-time and save the plot. The number of individual clusters is ordered by size, with Group 1 being containing the largest number of product ions. The graph layout, including the length of the lines within each SSN group, is automatically set by MATLAB and has no value for the interpretation of the SSN. For automatic annotation of the *m/z* values in the SSN based on user-selected ppm difference, the user can upload a database containing *m/z* values and known names of the corresponding ions. The annotated ions are identified in the list box by a green dot next to the *m/z* value. All SSN calculations and interrogations are supported by a GUI integrated into our i2i application (github: LanekoffLab),[4] and the functionalities are detailed in Fig. S5.

SSN analyses were performed on a standard Windows-based workstation (32 GB RAM), demonstrating that no specialized computational infrastructure is required.

## Immunostainings and tissue characterization

Fluorescent immunostainings were performed as previously published.[5] Briefly, frozen human tissue slides were dried at room temperature, fixed with 4% PFA at room temperature for 10 min, washed with PBS and incubated with blocking solution (10% normal species serum (NSS) in 0.05% Tween20 (Sigma-Aldrich)) for 30 min. Primary antibodies for PLP (1:300; Serotec, MCA839G), UEA-I (1:1000, Vector Labs B-1065), HLA-DR (1:500; Hybridoma) and Fibrinogen-FITC (1:300, Dako, F0111) were diluted in 1% NSS in 0.05% Tween20-PBS and applied on the slides overnight at 4 °C. Tissue sections were washed in PBS and incubated for 1 h with Alexa fluorophore-conjugated secondary antibody. Nuclear staining was performed using Hoechst fluorescent DNA stain (33258, Thermo Fisher Scientific) for 1 min. Images were acquired as a 20x overview scan at the Olympus VS200 slide scanner. Following, visual characterization was performed using QuPath software (version 0.4.4) to aid classification based on full (normal), diffuse (dirty appearing white matter) and absent (lesion) PLP immunoreactivity.[6]

## Regions of interest selection and data extraction

White matter brain tissue samples from three non-neurological control (NNC) and five people with multiple sclerosis (PwMS) were analyzed using one brain tissue section for each subject in a multimodal setting using silver-doped PA nano-DESI MSI, PIA, and immunohistochemical techniques. The stage of demyelination was determined using proteolipid protein (PLP) expression, where the absent PLP indicate most severe demyelination. The spatial information was transferred to the ion images where regions of interest (ROIs) were defined, stratified into normal PLP (n=19), diffuse PLP (n=20), and absent PLP (n=16). In particular, a region had to fulfil two criteria to be considered as an ROI: i) have a morphologically well-defined border from surrounding areas based on PLP and HLA-DR staining, ii) have a well-defined chemical profile based on ion images acquired using PA nano-DESI MSI. Morphologically distinct areas were defined according to the section “Immunostainings and tissue characterization”. Chemically distinct areas were defined by segmenting the PA nano-DESI MSI ion images based on the spatial distribution of monoacylglycerol, phosphatidylcholine, phosphatidylethanolamine, prostaglandin, free fatty acid and sterol lipid classes. For detailed information on ROIs, see Table S10

To compare the abundance of cholesterol and oxidized cholesterol products, average pixel intensities were extracted from each ROI using the i2i software[4] and the fractional abundance (FA) was calculated (Eq 3). In Eq. 3, the FA is calculated for each oxysterol based on its intensity over the sum of intensities of cholesterol (ST1) and all selected oxysterols (ST3, ST4, ST7).

$$FA = \frac{Int_{oxysterol}}{\sum(Int_{oxysterols}+Int_{cholesterol})} \qquad \textit{Eq. 3}$$

Subsequently, the cholesterol oxidation level was defined according to Eq 4, where FA is the fractional abundance of the molecule, and “ON” is defined by the additional oxygens in the sum-composition formula compared to cholesterol (0 for ST1, 1 for ST2, 2 for ST3 and ST4, and 4 for ST7).

$$Oxidation\ level = \sum(FA_i \times ON_i) \qquad Eq.\ 4$$

## Statistical analysis

Statistical analysis was performed, and boxplots were created using R v4.3.2 in RStudio.[7] The normality of the data was tested using the Shapiro-Wilk test. The pilot study cohort was balanced for age and post-mortem delay, and the covariate significance of the distinct patients was tested using a Kruskal-Wallis test. Since it was significant in only a part of the cases, we decided to perform non-parametric one-way testing but denote each donor by color codes as per Table S12. In multi-group comparisons, the Kruskal-Wallis test was performed, followed by Dunn´s post-hoc test, where a significant difference was indicated. For the two-group comparisons, the Wilcoxon test was used. FDR correction was performed in all analyses using the Benjamini-Hochberg method. Significant differences are indicated in the plots as follows. *:$p<0.05$, **:$p<0.01$, ***:$p<0.001$, ****:$p<0.0001$. The results of statistical tests are detailed in Table S12. In the boxplots, the black line denotes the median, the white dot the mean, the top and bottom edges of the box the interquartile range, and the whiskers extend to the minimum and maximum data points.

Hierarchical clustering-based heatmaps and Pearson correlation heatmaps and PLS-DA plots were created using MetaboAnalyst 6.0.[8] Missing data points were imputed by LOD (1/5 of the lowest intensity). For hierarchical clustering, values were Z-scored, Euclidean distance measure was set, and autoscaling was set based on samples.

# Step-by-step guideline for data acquisition and analysis

Here, a step-by-step guideline is provided to assist first-time users in setting up product ion alignment (PIA) and performing spatial similarity network (SSN) analysis. The SSN workflow is independent of the data acquisition strategy (parallel or sequential) and the ionization source. The only requirement for SSN analysis is the availability of $MS^2$ imaging ($MS^2I$) data, consisting of full-scan $MS^2$ spectra acquired for the precursor ion of interest at all pixels in the image.

1. **Mass spectrometer requirements for PIA.** PIA requires a tribrid mass spectrometer capable of parallel $MS^2$ acquisition. In this study, a Thermo Fisher Scientific Orbitrap IQ-X was used. On Thermo Fisher tribrid instruments (e.g., IQ-X), PIA can be enabled by disabling "MS1-only processing" in the diagnostics menu, allowing simultaneous $MS^2$ acquisition in the ion trap and Orbitrap mass analyzers.
2. **MSI sampling and ionization.** Any MSI sampling and ionization technique compatible with $MS^2$ imaging can be used. In this study, a pneumatically assisted nano-DESI (PA nano-DESI) source was employed. The construction and operation of the PA nano-DESI source are described in detail in the Experimental Section. The pixel size in the $x$-dimension is defined by the scan speed, as described in Figure 1c of the main manuscript, while the pixel size in the $y$-dimension is determined by the step size between adjacent scan lines.
3. **Data analysis software.** MSI data analysis was performed using the Ion-to-Image (i2i) software, available at https://github.com/LanekoffLab/i2i. System requirements and installation instructions are provided in the repository.
4. **Data format and SSN parameters.** The i2i software requires input data in *.mzML* format. After loading the data, the user selects the appropriate scan filter and similarity metric (SSN, mean squared error, or cosine similarity). Suggested parameters for similarity threshold, mass tolerance (ppm), and minimum and maximum intensity thresholds can be altered by the user to match the experiment.
5. **Product-ion library.** A product ion library can be provided to annotate the m/z in the clusters. Product-ion libraries used in this study are provided in Tables S2–S4. Only an Excel file containing the list of *m/z* values and corresponding annotations is required. The m/z in the cluster that matches the library are highlighted with a green marker to assist annotation (see Figure S5).
6. **SSN construction.** SSN clustering is initiated by clicking the "Run" button in the SSN module of the i2i software. Product ion images are clustered into spatial similarity networks based on the selected similarity metric.
7. **Visualization of SSN results.** Ion images corresponding to individual SSN clusters are displayed in the "*m/z*" sub-tab of the SSN user interface, while the complete SSN is visualized in the "Network" sub-tab (see Figure S5).
8. **Spectral validation.** Deconvoluted $MS^2$ spectra derived from SSN clusters can be exported and compared with online spectral libraries or authentic standards for further validation. An example comparison with a PC(34:1) standard is shown in Figure 3h.

# List of supplementary tables

**Table S1** Level 5 annotations of targeted mass channels based on accurate mass match

**Table S2** Fragmentation library for PE lipids based on characteristic and diagnostic product ions of $[M+Na]^+$ parent ions.

**Table S3** Fragmentation library for PE plasmalogens based on characteristic and diagnostic product ions of $[M+Na]^+$ parent ions.

**Table S4** Fragmentation library for PC lipids based on characteristic and diagnostic product ions of $[M+Na]^+$ parent ions.

**Table S5** Acyl chain-specific annotation (after HCD fragmentation of sodiated adducts) of glycerophospholipid species in mouse brain tissue using PIA SSN.

**Table S6** Validation of annotated glycerophospholipid species in mouse brain tissue using PIA SSN with different experimental settings. (n.t.: non-targeted and no overlap with targeted isolation windows, n.d.: characteristic product ion not detected)

**Table S7** Detailed parameter list for PA nano-DESI MSI including full target lists for all the experiments performed using PIA SSN.

**Table S8** Suggested annotation levels and their description for MSI studies.

**Table S9** List of diagnostic product ions (DPIs) for cholesterol and the investigated oxidized cholesterol metabolites.

**Table S10** Medical details of human subject samples used for the multimodal characterization of cholesterol oxidation in the human multiple sclerosis brain.

**Table S11** List of SSN parameters used for the generation of the networks.

**Table S12** Results of statistical tests during the characterization of cholesterol oxidation in the human multiple sclerosis brain.

*The supplementary tables are supplied as one separate Excel file.*

# Supplementary figures

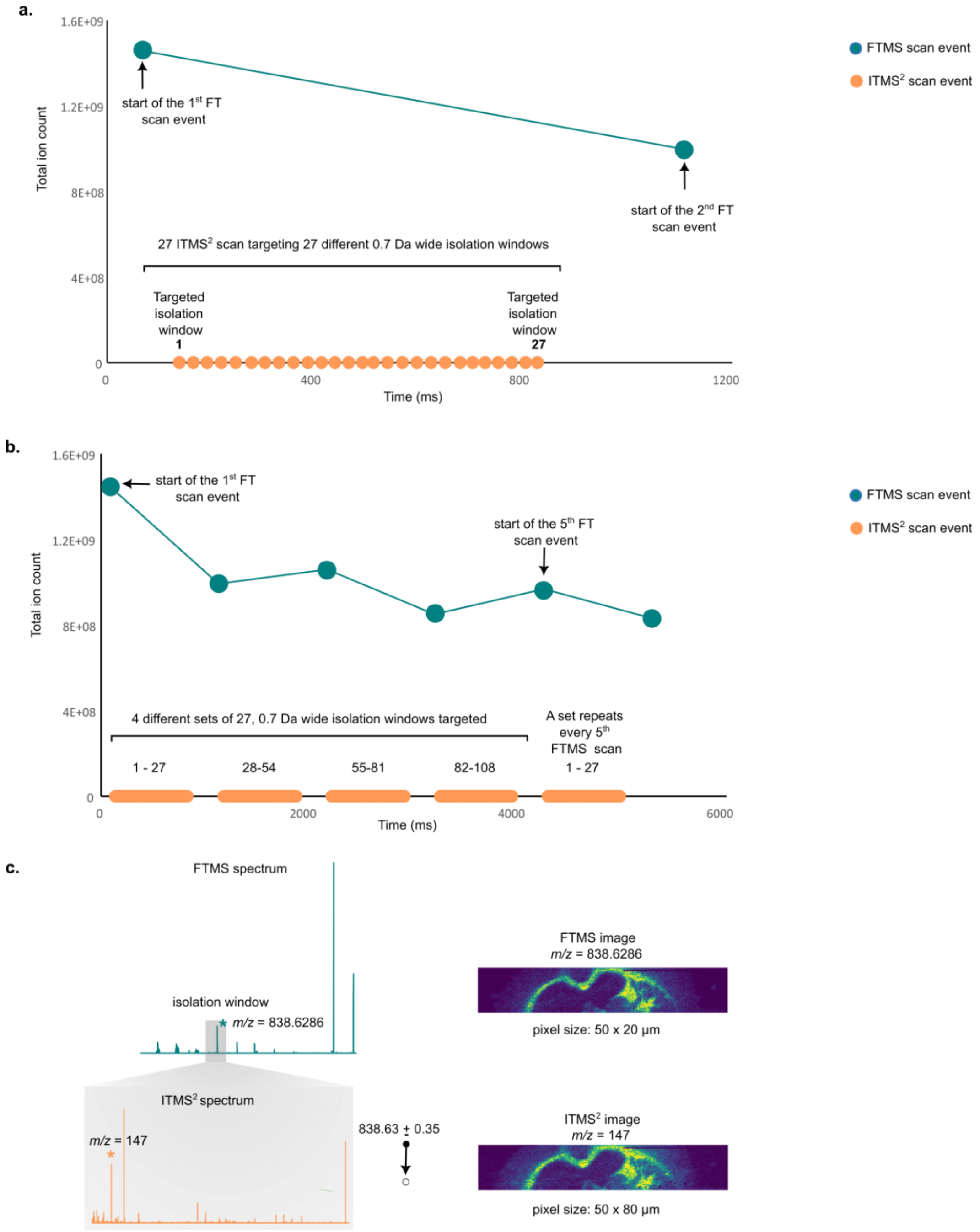


**Figure S1 Parallel image acquisition (PIA) enables multiplexed MS$^2$ imaging without compromising acquisition time**

Total ion current (TIC) plotted against time illustrates the sequence of events during one complete FTMS scan at resolution 500K (*m/z* =200) acquired using PIA. PIA enables sequential fragmentation of 27 targeted isolation windows (0.7 Da width) using ITMS between two high-resolution FTMS scans. This allows MS$^2$ spectra to be acquired in parallel with full scan MS. **(b)** PIA is scalable by looping through multiple inclusion lists. Here, four sets of 27 isolation windows (totaling 108) are fragmented between 4 FTMS scans, significantly increasing molecular coverage. **(c)** Looping increases the pixel size of ITMS$^2$ data by a factor equal to the number of loops (e.g., 4× for four lists) while FTMS pixels remain unchanged. For example, the ion image of a mouse brain section with FTMS for precursor ion at *m/z* = 838.6286 has a pixel size of 50 x 20 µm, while its corresponding product ion in ITMS$^2$ at *m/z* = 147 has a pixel size of 50 x 80 µm.

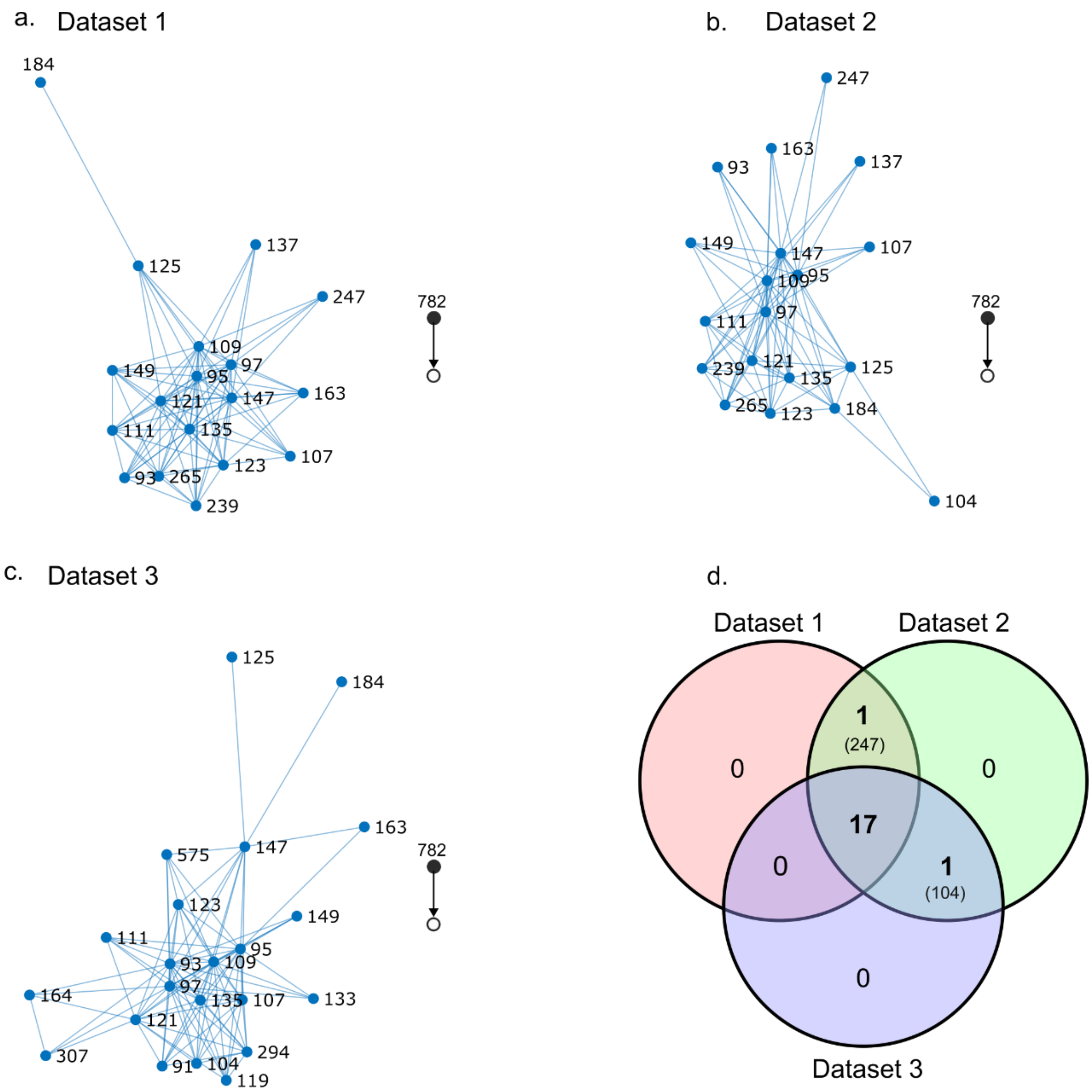


e.

| Comparision | Jaccard Similarity | Formula |
|---|---|---|
| Multi dataset (1,2,3) | 0.895 | I1∩2∩3I/I1U2U3I |
| Pairwise dataset (1-2) | 0.947 | I1∩2I/I1U2I |
| Pairwise dataset (1-3) | 0.944 | I1∩3I/I1U3I |
| Pairwise dataset (2-3) | 0.947 | I2∩3I/I2U3I |

**Figure S2 Comparison of fragment-ion clusters across three MS$^2$I datasets.**

**(a–c)** Clustered networks of fragment ions for precursor *m/z* 782 from Datasets 1, 2, and 3 using cosine similarity. Cosine similarity is independent of absolute intensity; therefore, it is useful for comparing datasets or for comparing data obtained under different experimental conditions. A threshold of 0.4 was applied, representing the maximum allowed deviation between fragment ion intensity patterns **(d)** Venn diagram summarizing the overlap of fragment ions across the three datasets. A total of 19 unique product ions were detected across all three datasets, with 17 product ions common to all datasets indicated in the center. **(e)** Table with multi-dataset and pairwise Jaccard similarity values, quantifying the reproducibility of the clustering. Formulas for each Jaccard index are provided in the third column. These similarity values indicate strong agreement between fragment-ion clusters obtained from different datasets.

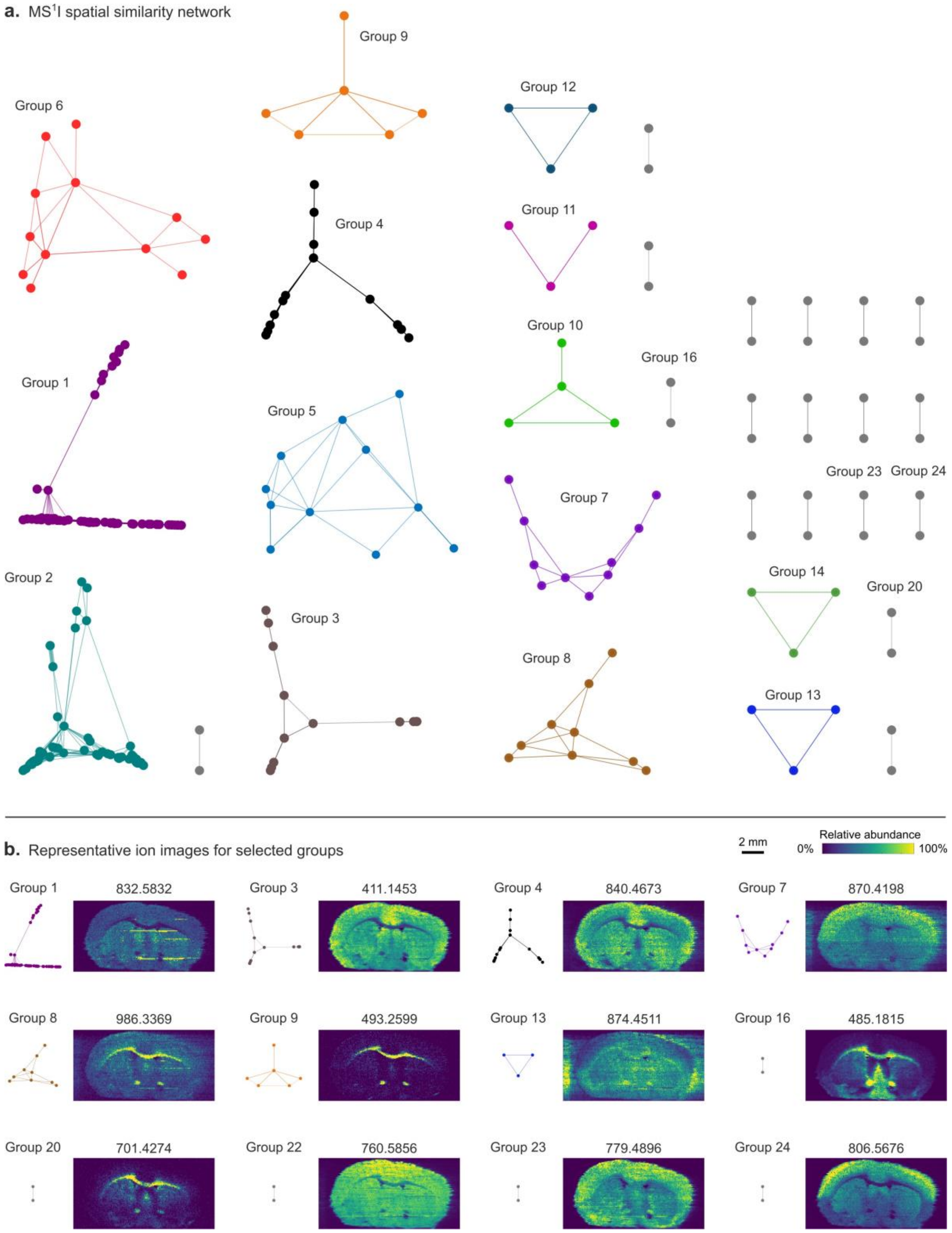


**Figure S3 The SSN tool enables non-targeted data exploration of MS$^1$I data**

The SSN is not limited to using MS$^2$ data and product ions. On the contrary, SSN also rapidly clusters molecular ions with similar distribution, despite the increased number of available *m/z* values in the 200-2000 range. The SSN for a MS$^1$I data set displays a large complexity of spatial distributions **(a)**. Altogether, the network analysis of 272 *m/z* values was performed, where all had at least one edge connecting to the node of another *m/z* feature. The parameters were 5 ppm mass tolerance, 0.15 error limit, 15000 intensity limit, SSE distance metric. Further 760 *m/z* values represented by disconnected nodes were discarded from the analysis. Representative ion images of separated groups using the SSN network **(b)** show the variety of spatial distributions of mouse brain lipids. All ion images were normalized to TIC, and intensities were scaled to the 99$^{th}$ percentile. For experimental details, see Dataset 4 in Table S7, for SSN details, see Table S11.

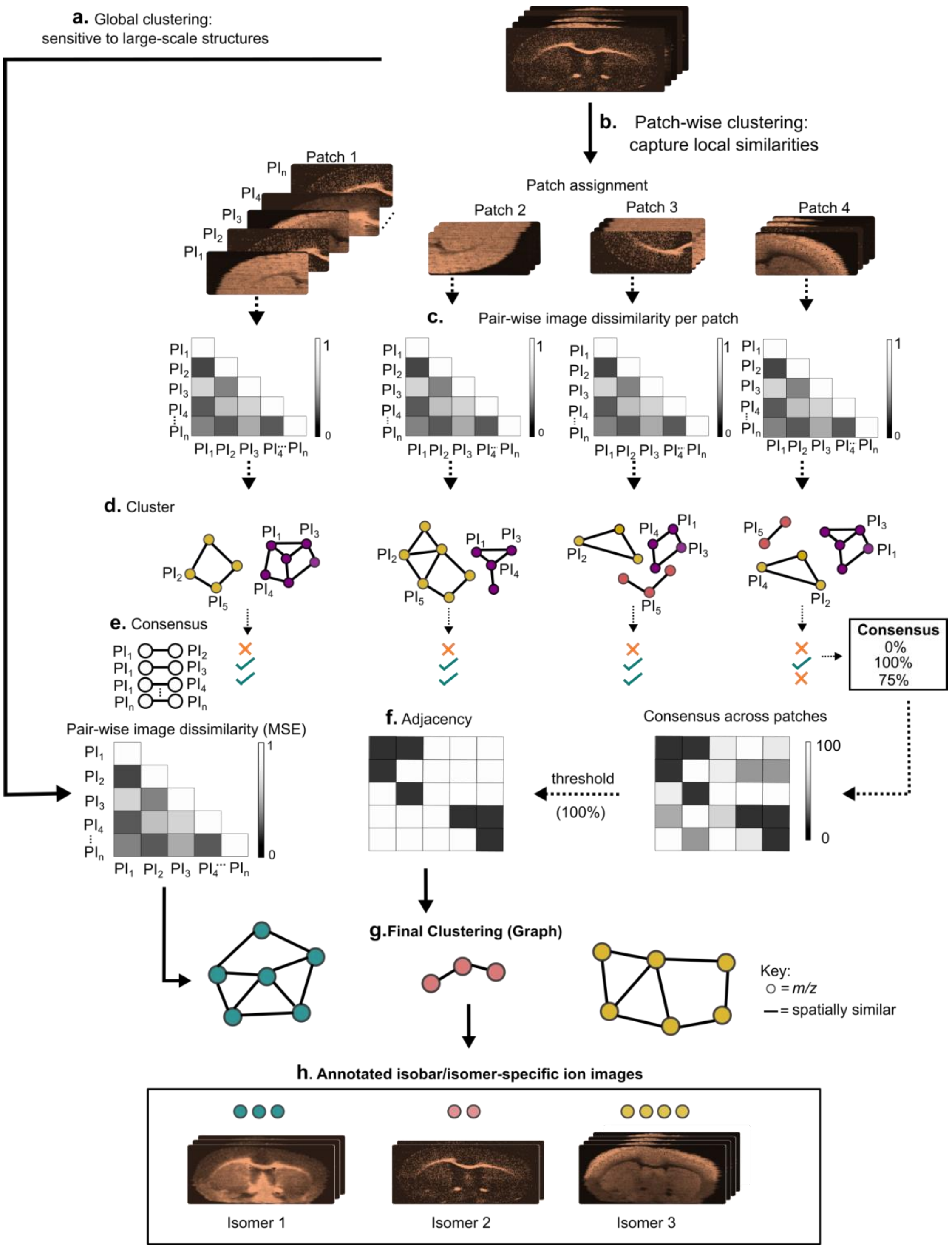


**Figure S4 Schematics of patch-wise coherent spatial similarity networking (coherent SSN)**

**(a)** Global SSN network as described in Figure 2. b-i, patch-wise clustering workflow. **(b)** The image is divided into equal-sized patches where the number of patches can be 4, 9 or 16. **(c)** Pairwise spatial similarity is computed between product ion images using either SSE, MSE or cosine similarity. **(d)** Product ions in each patch are clustered using a connected components algorithm based on spatial similarity. **(e)** A pair-wise consensus is taken for all ion images across clusters arising from different patches, resulting in a consensus matrix. For example, if a pair of ion images is connected across all clusters, then the consensus for that pair is 100%. **(f)** An adjacency matrix is created from the consensus matrix by filtering out ion image pairs above a specified threshold. If the threshold is 100%, the ion image pairs that are connected in all clusters are filtered. **(g)** Product ions are finally clustered using the connected components algorithm based on the adjacency matrix, and thus, coherent spatial similarity across patches. **(h)** Each spatially coherent cluster is matched against a curated in-house product ion library to propose molecular identities.

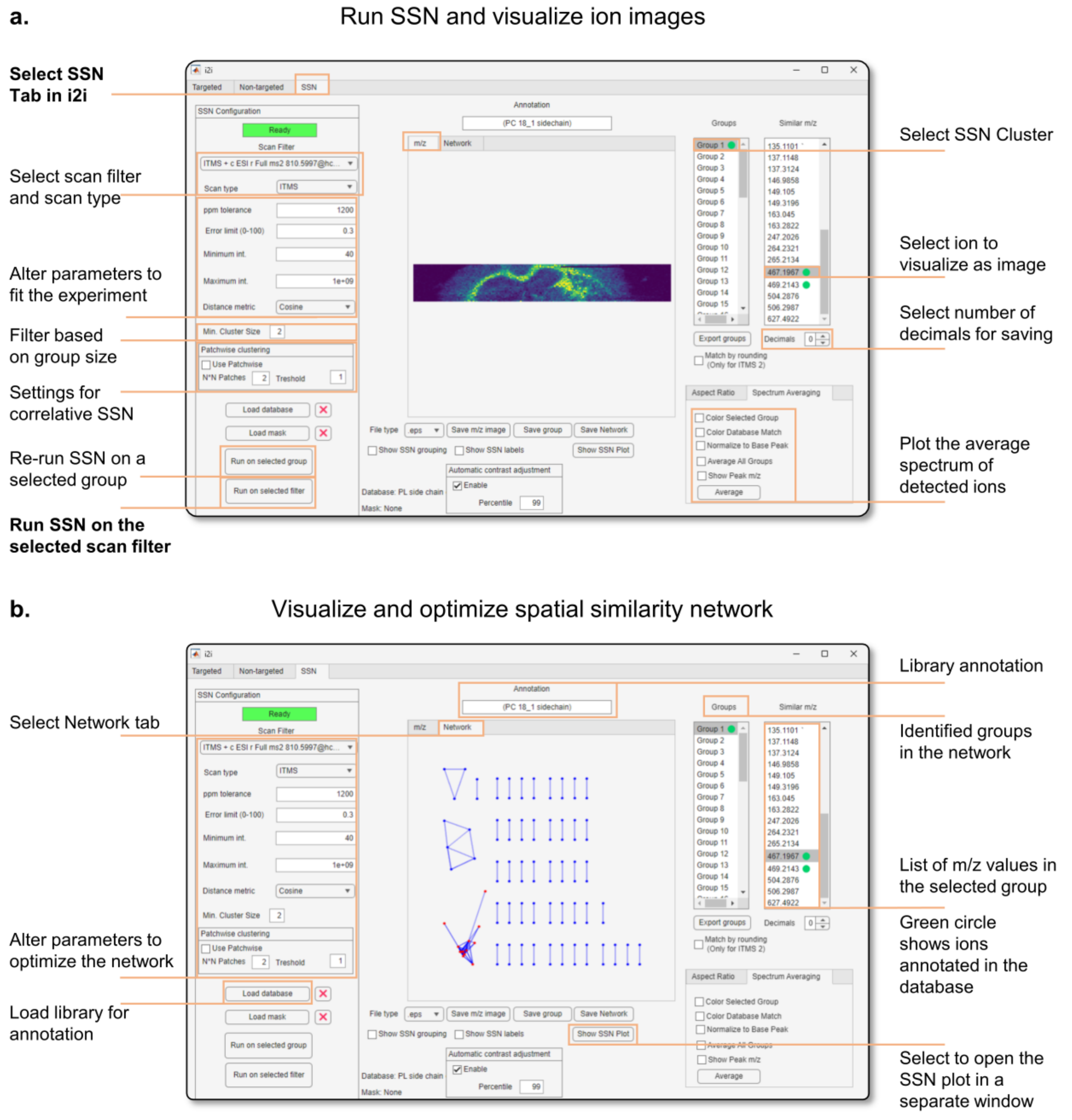


**Figure S5 Graphical user interface of the new SSN module in i2i**

First, the scan filter and scan type are selected based on the loaded data, and data extraction parameters are defined. Default parameters are suggested, although optimization may be required for each scan filter depending on the complexity and intensity of the detected ions **(a)**. The SSN automatically clusters ions in the selected scan (here, FTMS, $FTMS^2$ or $ITMS^2$) into networks based on either the sum of squared error (SSE), the mean of squared error (MSE) or the cosine spatial similarity metric of the ion distributions. The network group and *m/z* of the ion image to be displayed are selected together with the relevant number of decimals or rounding, which can be selected depending on the mass resolution. The product ion image in the *m/z* tab shows the acyl chain 18:0 (*m/z* 467.2 of $[PC(36:1)+Na]^+$ (*m/z* 810.2) with the annotated ions from the loaded database marked with a green dot. In the network tab, the resulting SSN is displayed **(b)**. The network shows nodes of *m/z* values that are clustered based on their spatial distributions. The SSN groups are ordered by size and the graph layout is automatically set by MATLAB. To interrogate the SSN, the user can select groups and their *m/z* values in the list box next to the displayed SSN. Alternatively, by opening the SSN as a new window, the user can hover over the displayed SSN to view information on each node and save the plot. For the automatic annotation of *m/z* values in the SSN, the user can upload a database containing *m/z* values and the corresponding known names of the ions for matching (Tables S2-S4). For experimental details, see Dataset 1 in Table S7, for SSN details see Table S11.

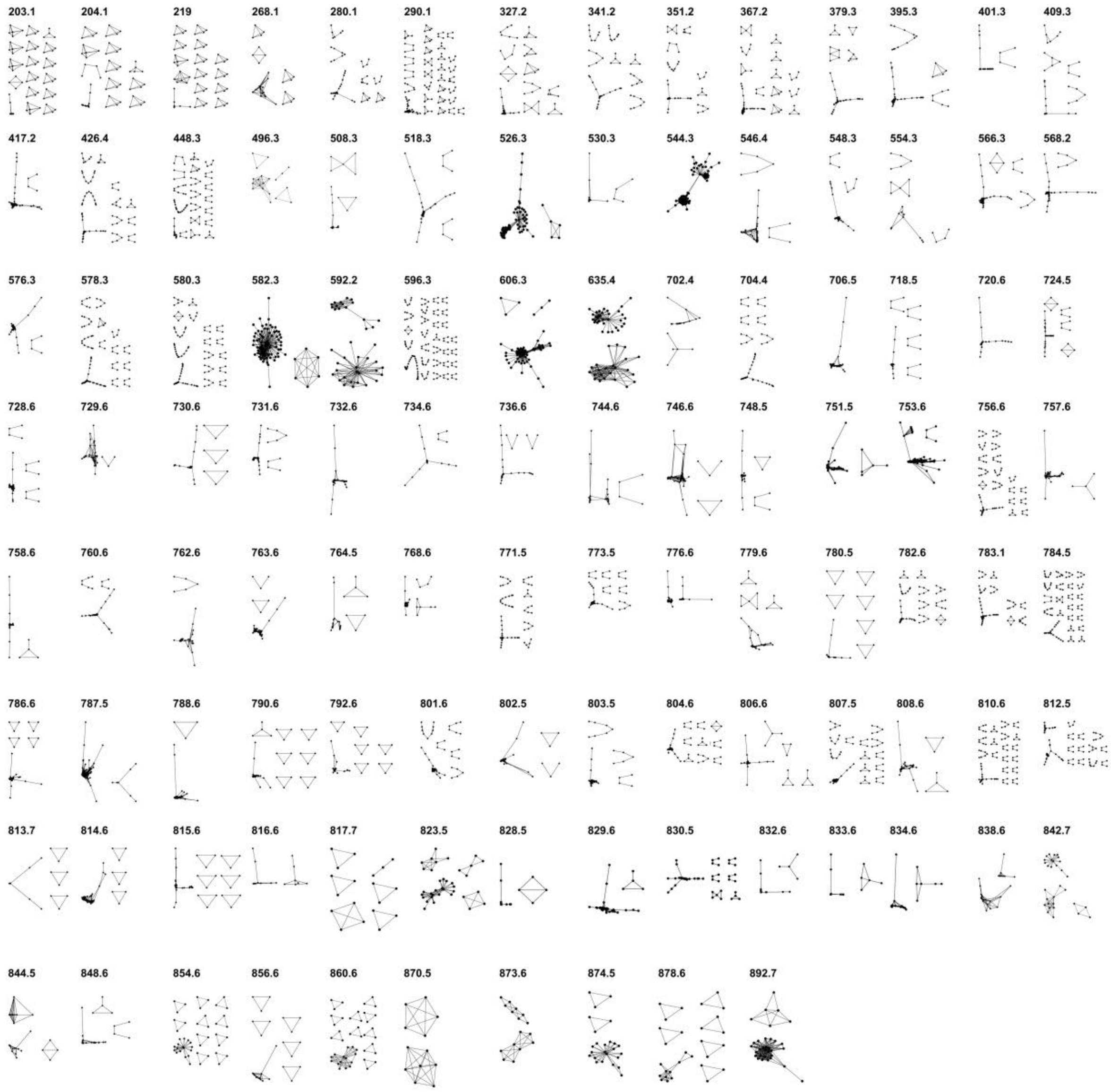


**Figure S6 SSN of 108 targeted mass channels show a great versatility of spatial distributions**

Data from PIA (N=4, n=27) were used for the SSN. Note that the parameters were not optimized for each scan filter, instead the parameters were kept the same at: 800 ppm tolerance, and 5 error limit for all scan filters. The m/z values of the targeted precursor ions are shown on top of the respective SSN. For experimental details see Dataset 1 in Table S7, for SSN details see Table S11.

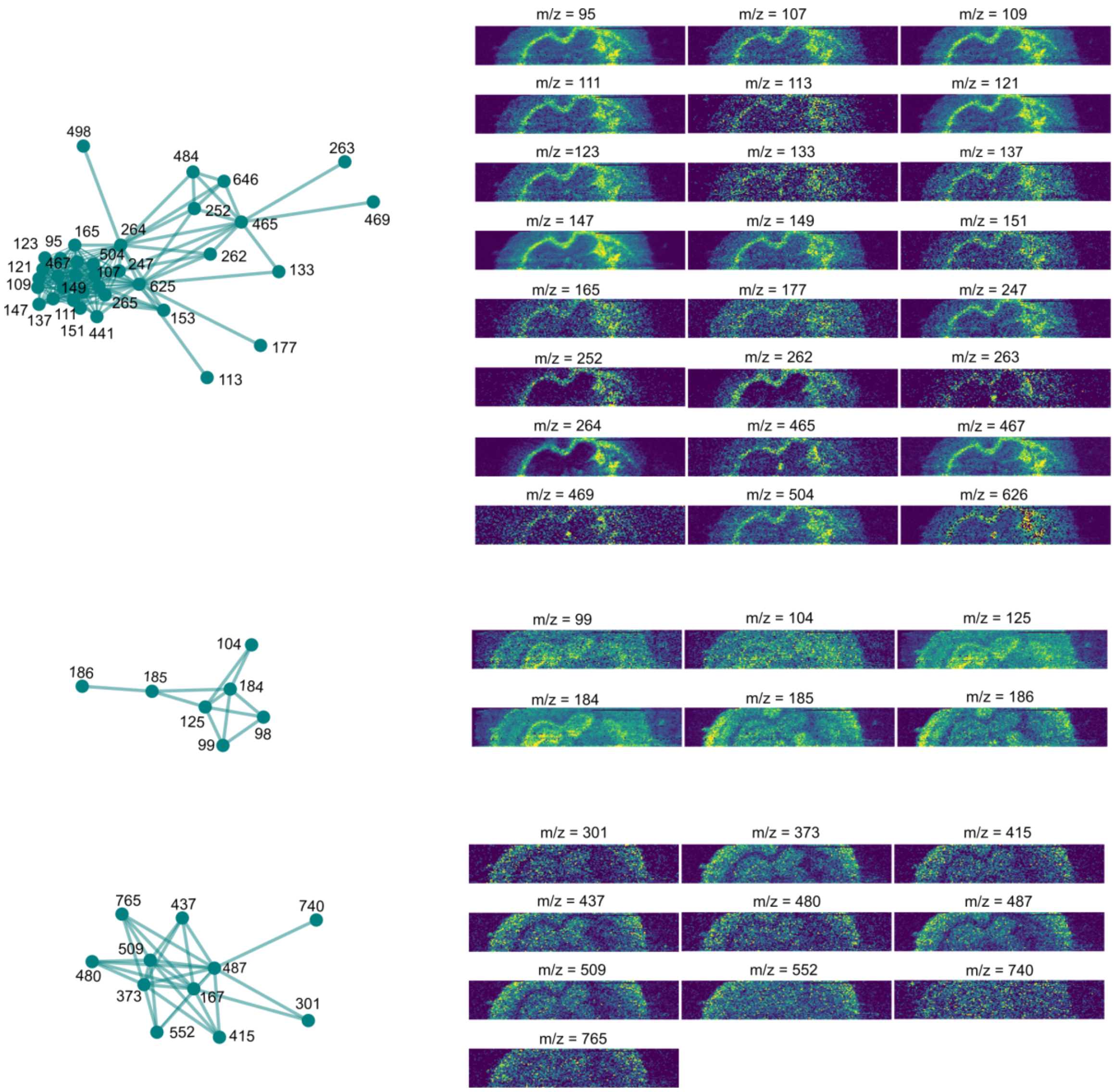


**Figure S7 Product ion network clusters and corresponding images from fragmentation of the isolation window centered at 808.60 ± 0.35 Da**

The product ion images were acquired using the PIA of a mouse brain tissue section. The subsequent SSN reveals that product ions are separated into three groups based on their spatial distribution, suggesting the presence of at least three structurally different precursors. For experimental details see Dataset 1 in Table S7, for SSN details, see Table S11.

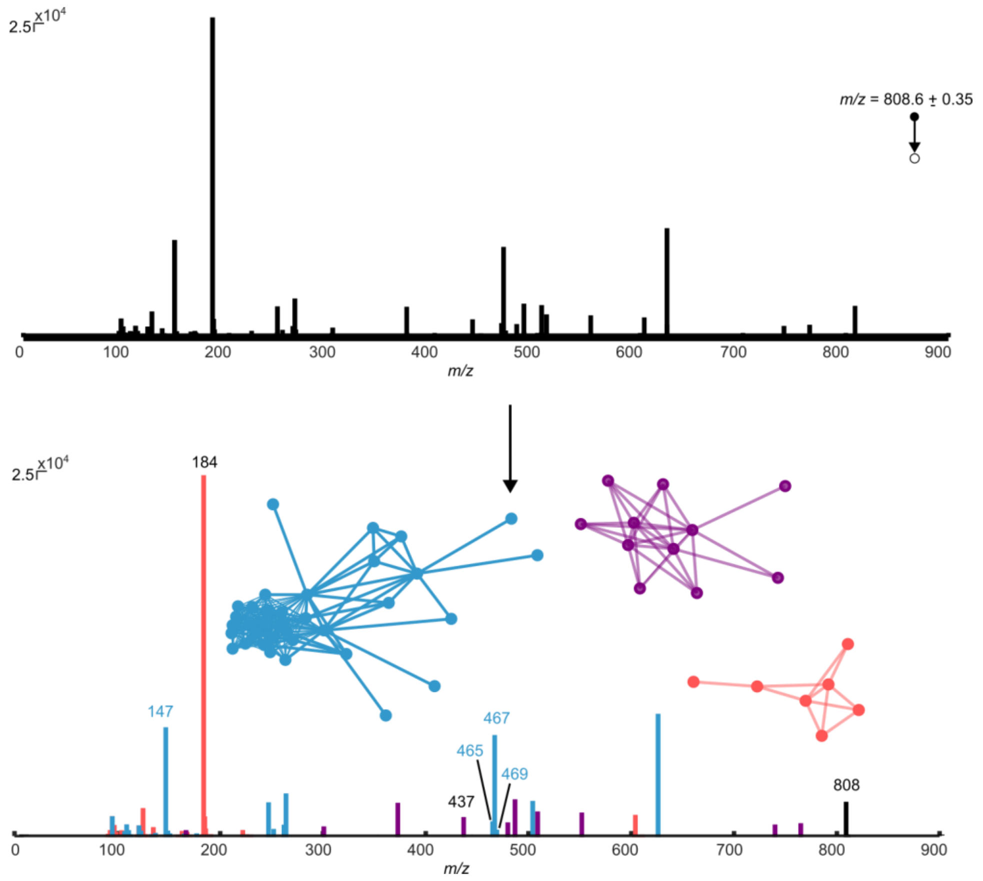


**Figure S8 SSN works as a separation dimension unique to MSI and deconvolutes product ion mass spectra**

The convoluted $ITMS^2$ mass spectrum produced from the product ions of all precursors in the 808.6 ± 0.35 window is readily deconvoluted by SSN. The product ions network clusters shown in blue, purple, and red correspond to the blue, purple, and red product ions in the deconvoluted mass spectrum, respectively. For experimental details see Dataset 1 in Table S7, for SSN details, see Table S11.

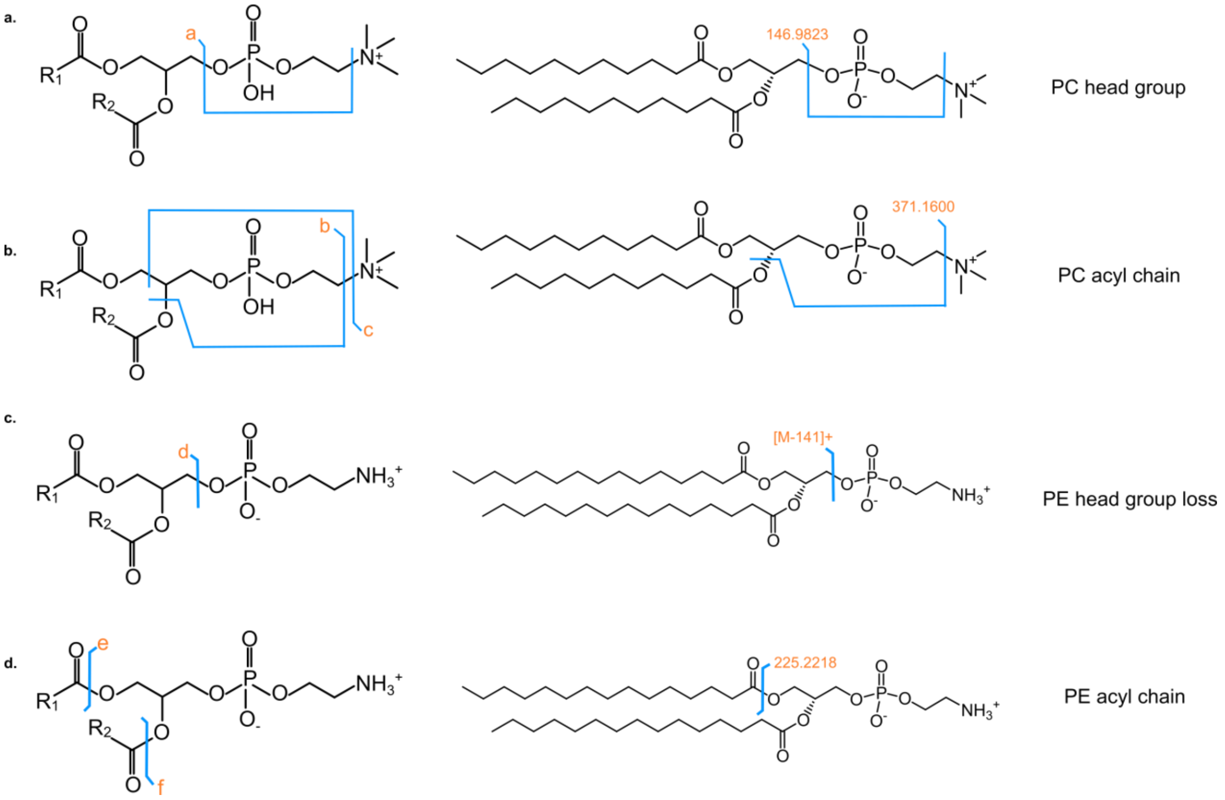


**Figure S9 Fragmentation patterns of phosphotidylcholine (PCs) and phosphotidylethanolamine (PEs) as sodiated adducts in HCD**

**(a)** The PC head group is detected at m/z 146.9823 after to loss of the acyl chains and the choline group. **(b)** After the loss of choline and one acyl chain, either the sn-1 or sn-2, the *m/z* of the remaining head group, the glycerol backbone and the remaining acyl chain is detected. **(c)** The neutral loss of the PE head group (-141) results in the detection of the acyl chains and the glycerol backbone. **(d)** Either the sn-1 and sn-2 acyl chains of the PE are detected after the fragmentation in the ester linkage. Note that both PC and PE fragmentation in HCD produce diagnostic product ions corresponding to the acyl sidechain as well as information on head group loss in positive ion mode. Contrarily, collision induced dissociation (CID) only provides information on head group loss.

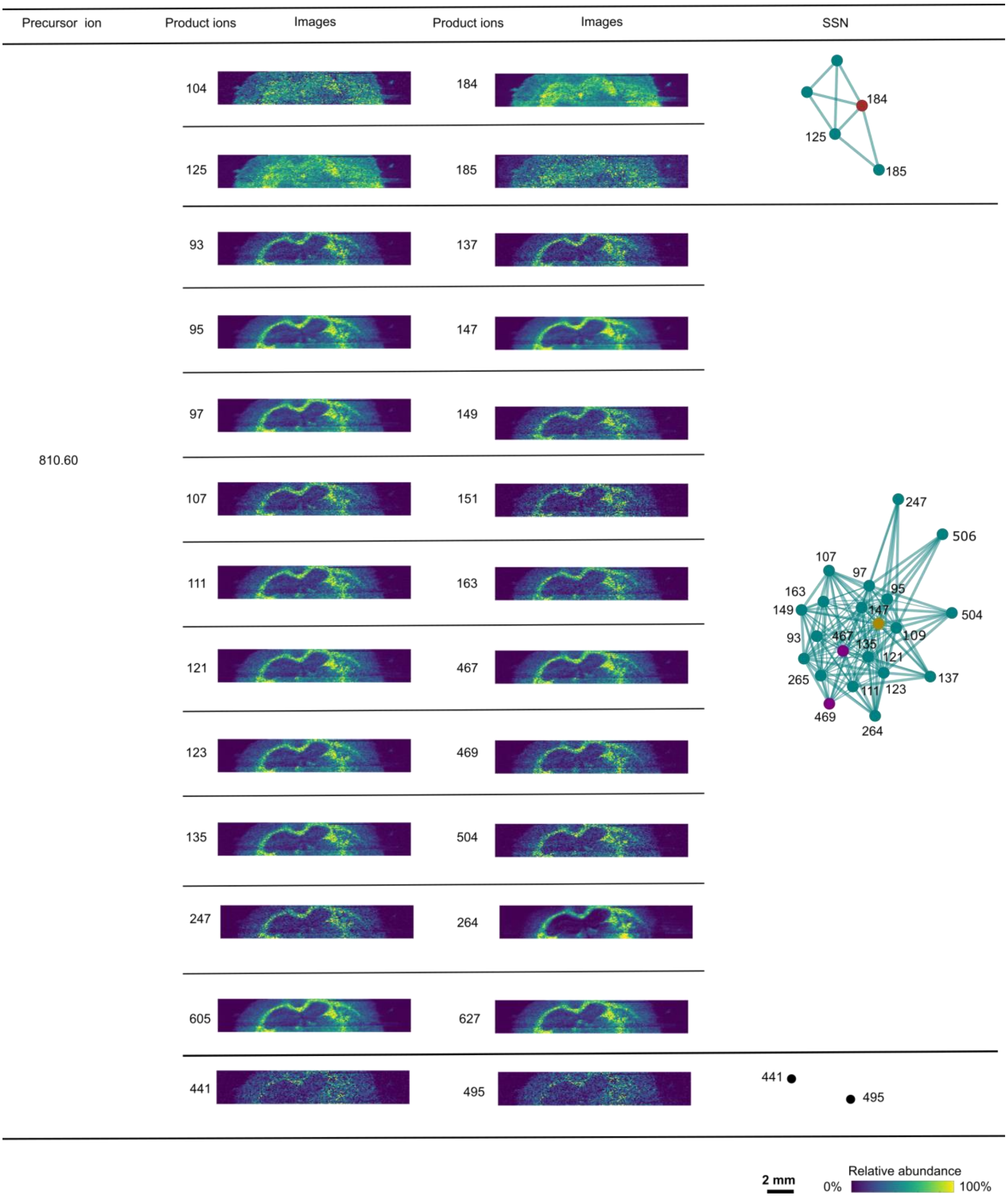


**Figure S10 Product ion image networks from PIA SSN**

The *m/z* values and corresponding product ion images were acquired from the precursor window of 810.60 ± 0.35 Da using the PIA of a mouse brain tissue section. The subsequent SSN reveals that product ions are separated into two groups based on their spatial distribution, suggesting the presence of two structurally different precursors. Two additional disconnected nodes are present, indicating the presence of a third, but less abundant, precursor. From top to bottom, the represented ions are [PC 38:4+H]$^+$, [PC 18:0_18:1+Na]$^+$, and [PC 16:0_20:1+Na]$^+$. For experimental details see Dataset 1 in Table S7, for SSN details, see Table S11.

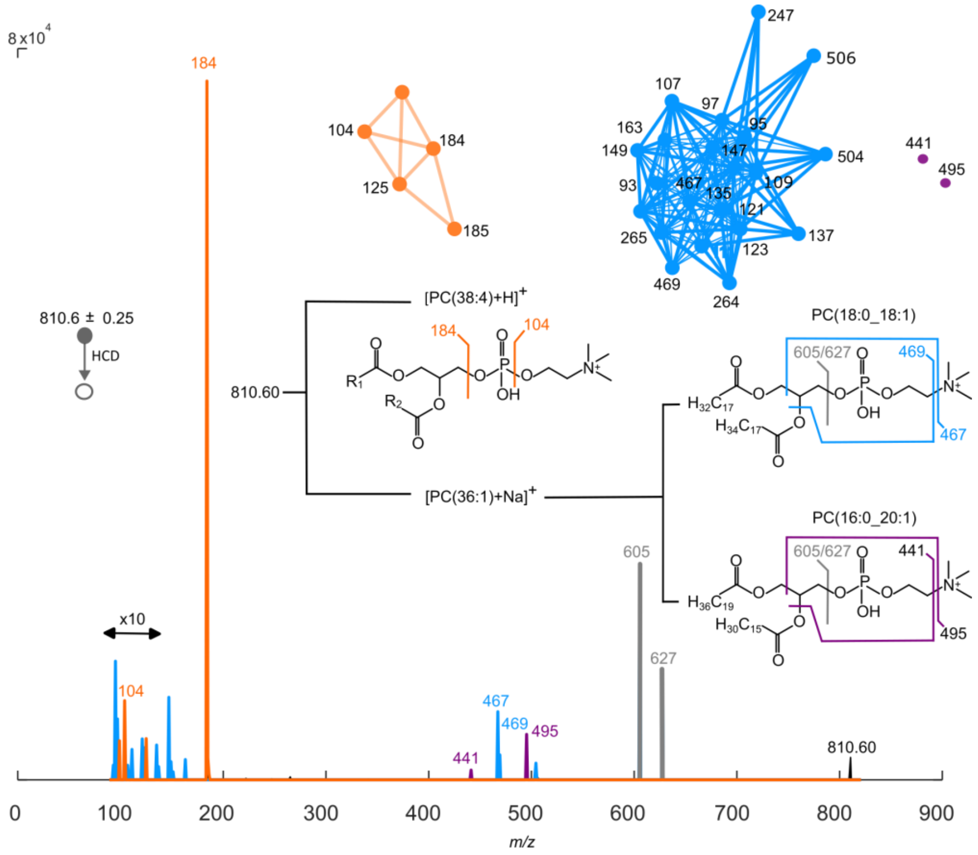


**Figure S11  SSN deconvolutes product ion mass spectra and facilitates annotation of isobars and isomers**

The convoluted ITMS$^2$ mass spectrum produced from the product ions of all precursors in the 810.6 ± 0.35 window is readily deconvoluted by SSN. The product ions corresponding to the blue and orange SSN are shown in blue and orange, respectively. This shows that the two distinct isobars of $[PC(38:4)+H]^+$ and $[PC(36:1)+Na]^+$, which have different spatial distributions, both originate from the precursor ion at *m/z* 810.60. Furthermore, the data reveals that $[PC(36:1)+Na]^+$ consists of the two isomers PC(18:0_18:1) and PC(16:0_20:1). For experimental details see Dataset 1 in Table S7, for SSN details see Table S11.

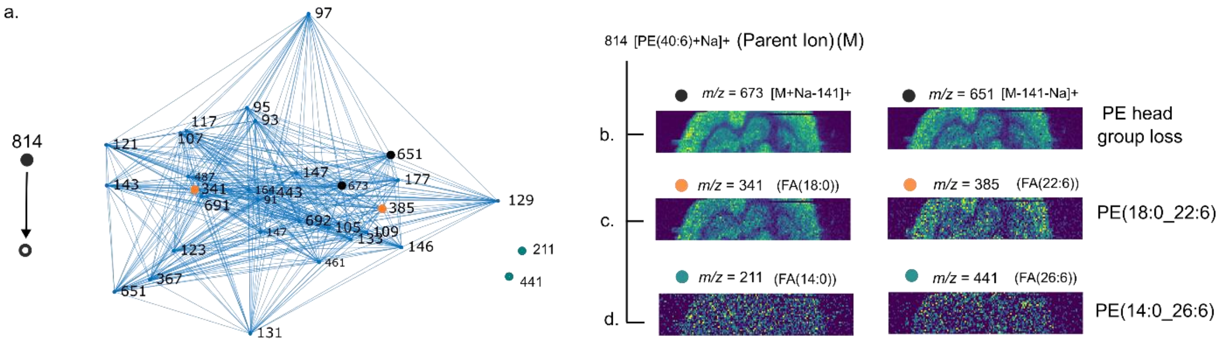


**Figure S12 Annotation of phosphatidylethanolamine species [PE(40:6)+Na]⁺ by SSN.**

**(a)** The SSN of product ion images derived from the fragmentation of *m/z* = 814.5 results in one cluster and two singletons (211 and 441) using MSE similarity and an error threshold of 10. **(b)** Product ion images related to head group loss indicate a sodiated adduct of phosphatidylethanolamine species. **(c)** Product ions related to fatty acid acyl chain FA(18:0) and FA(22:6). **(d)** Fatty acid acyl chains FA(14:0) and FA(26:6) have an error greater than 10% likely due to being sparsely distributed and does not cluster at these settings. The combination shows that There are two isobars belonging to PE(40:6): PE(18:0_22:6) and PE(14:0_26:6).

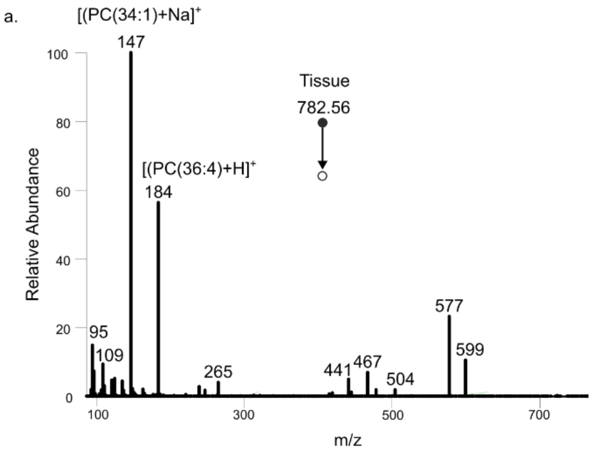


**Figure S13 Chimeric MS2 spectra obtained from fragmentation of m/z 782.56.**
(a) Chimeric $MS^2$ spectrum obtained directly from tissue following fragmentation of *m/z* 782.56, corresponding to co-isolated $[PC(34:1)+Na]^+$ and $[PC(36:4)+H]^+$ species.

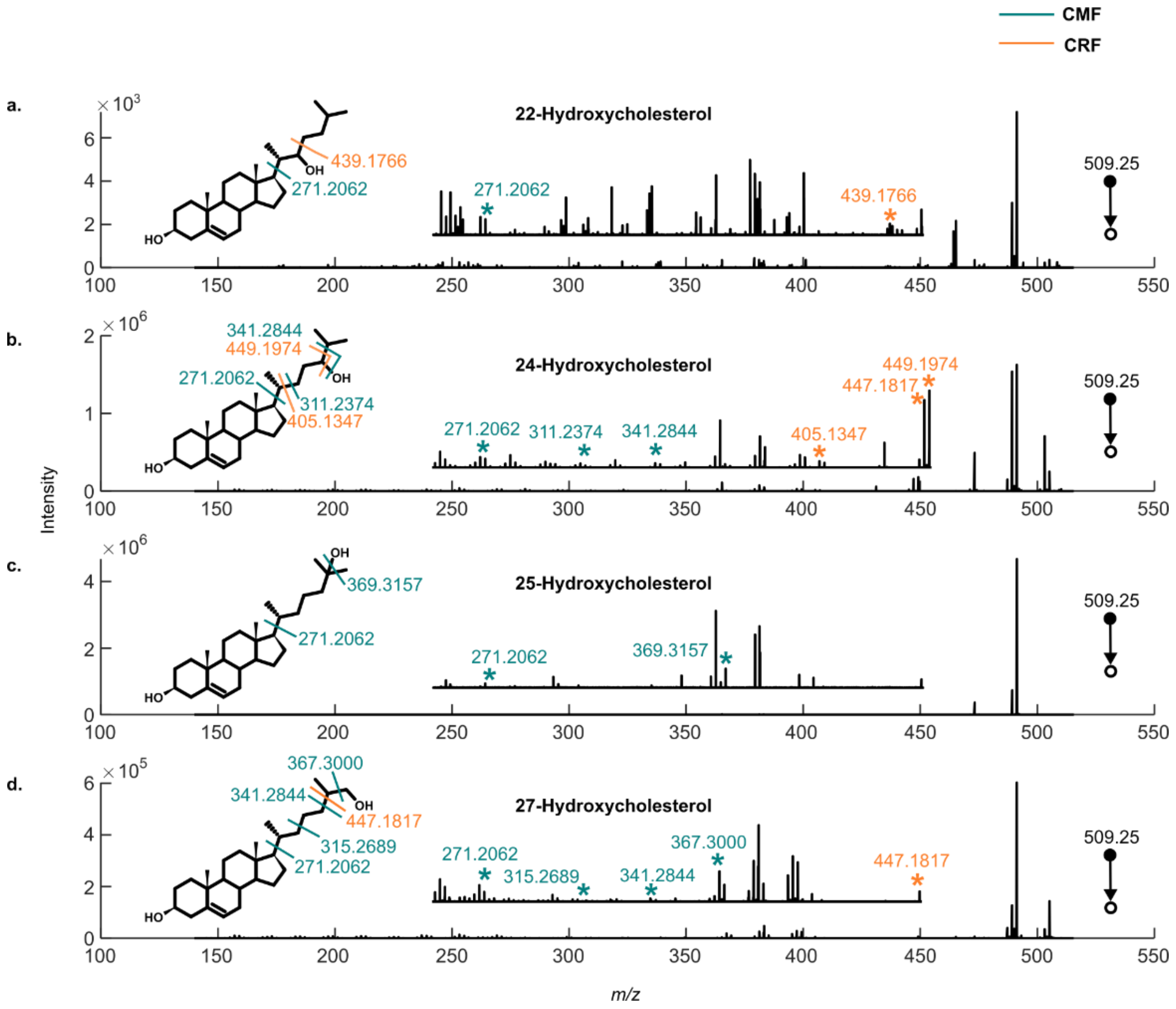


**Figure S14 Fragmentation of argenated adducts of hydroxycholesterols**

Consistent fragmentation sites are identified for argenated hydroxycholesterols upon HCD. $FTMS^2$ mass spectrum of 5 µM standards of **(a)** 22-hydroxycholesterol, **(b)** 24-hydroxycholesterol, **(c)** 25-hydroxycholesterol, and **(d)** 27-hydroxycholesterol. The annotated product ions are generated by either charge migration fragmentation (CMF, in blue) or charge retention fragmentation (CRF, in orange). In CRF $Ag^+$ is retained on the product ions while in CMF $Ag^+$ is lost during fragmentation.

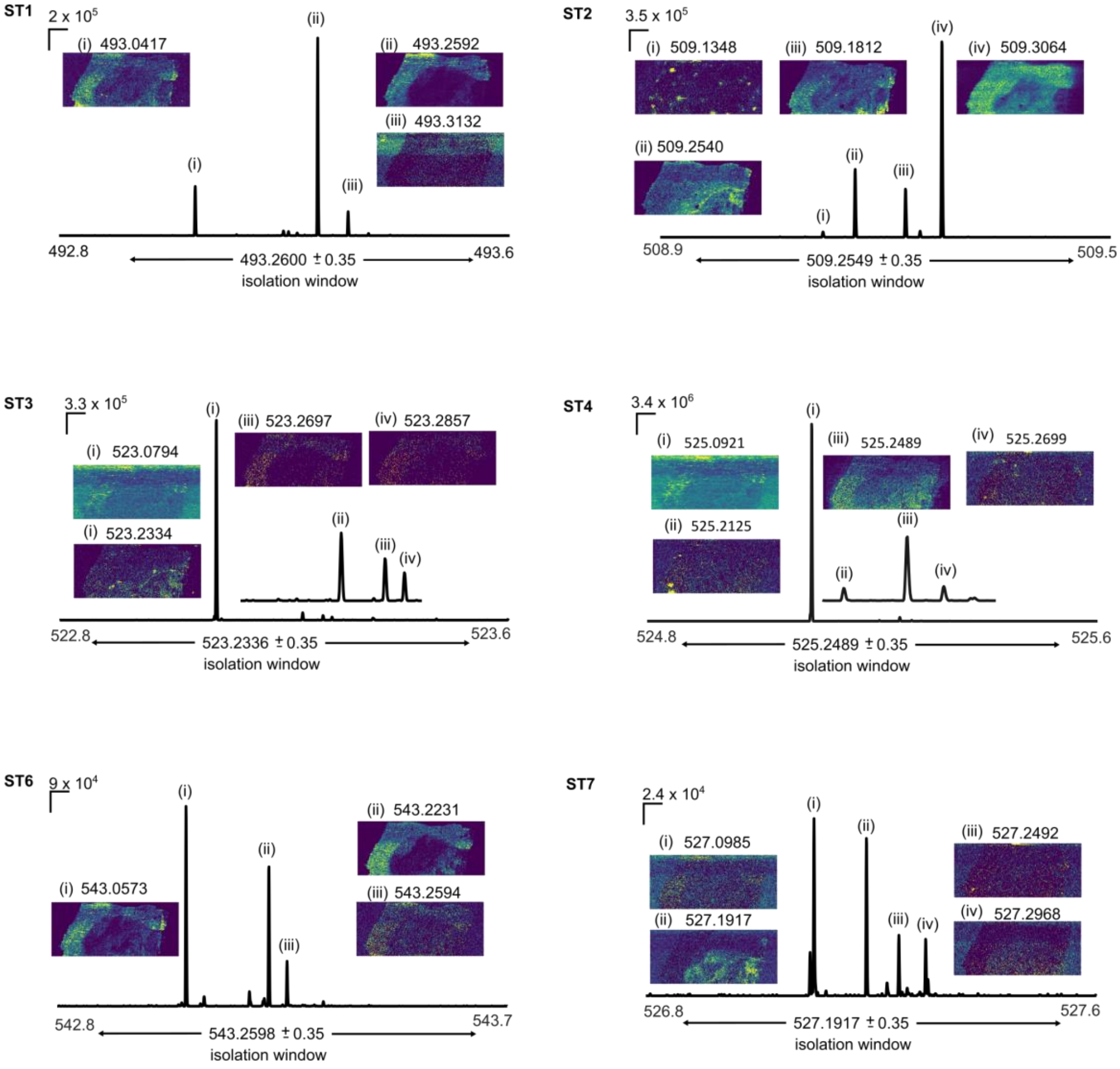


**Figure S15 The isolation window contains multiple precursor ions**

FTMS spectra detailing the isolation window of precursor ions selected for ITMS$^2$. FTMS$^1$ spectra are displayed for the isolation windows for cholesterol and oxidized cholesterol products (ST1-4 and ST6-7). Data is acquired from in human multiple sclerosis brain tissues and show the high complexity of detected ions in the selected regions. The ion images show the different distributions that arise from the respective precursors (i-iv) in the isolation windows. The co-isolation of multiple precursors increases complexity when decoding ITMS$^2$ spectra. For experimental details see Dataset 5 in Table S7.

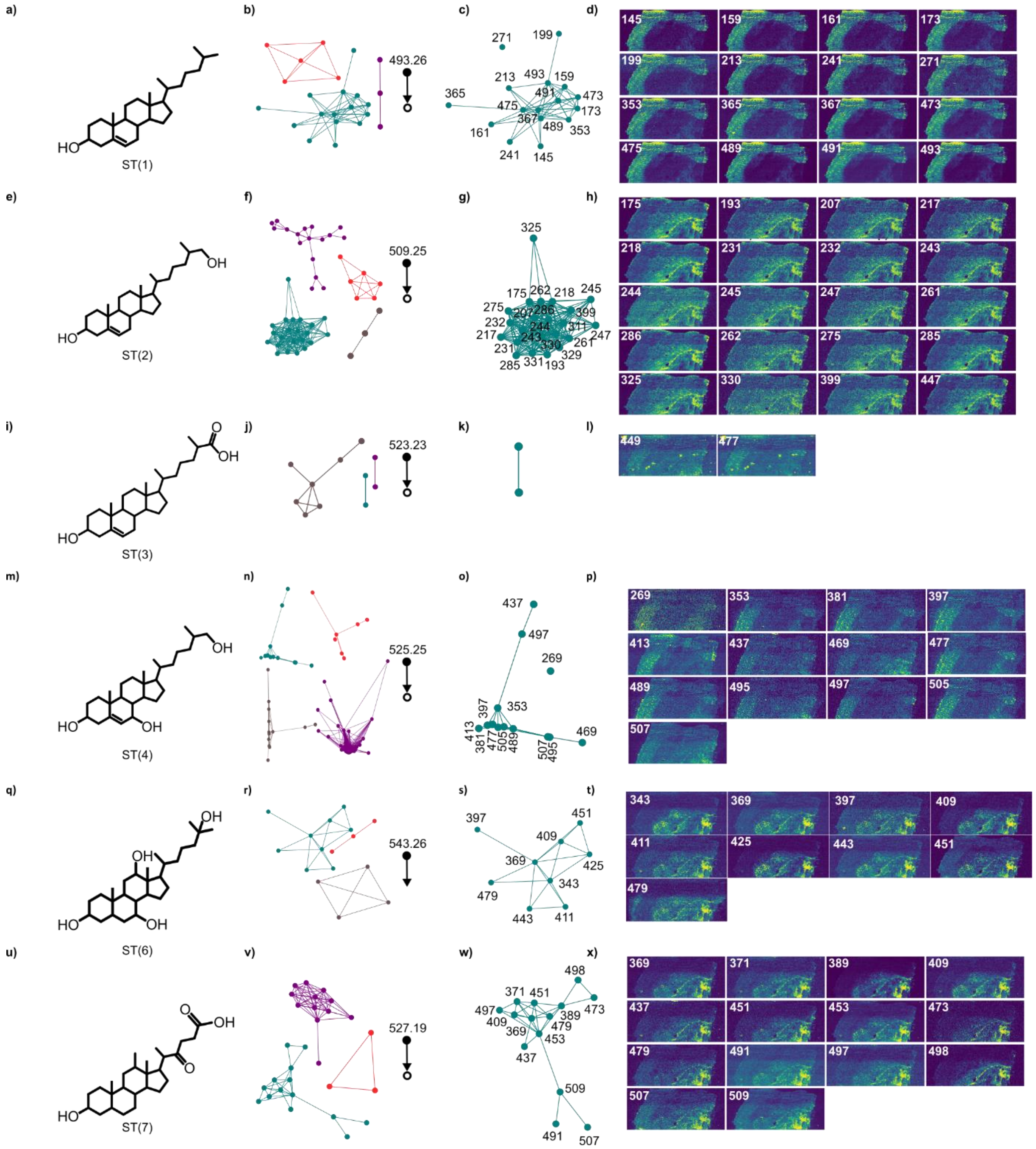


**Figure S16 The SSN deconvolutes complex ITMS$^2$ spectra produced by the fragmentation of co-isolated precursor ions**

Characterization of oxysterol structures detected by PA nano-DESI MS$^2$I of human multiple sclerosis brain tissue. Structural annotation of cholesterol and oxidized cholesterol products using HCD of $[M+^{107}Ag]^+$ adduct in ITMS$^2$. **(a)-(d)** cholesterol, **(e)-(h)** hydroxycholesterol, **(i)-(l)** hydroxycholestenoic acid, **(m)-(p)** dihydroxycholesterol, **(q)-(t)** cholestantetraol, **(u)-(x)** homodeoxycholic acid. For each targeted molecule, SSN and all product ions in the SSN are provided. One additional structural annotation (dihydroxy-cholestenoic acid) is shown in *Figure 4* of the main text. For experimental details, see Dataset 5 in Table S7, for SSN details, see Table S11.

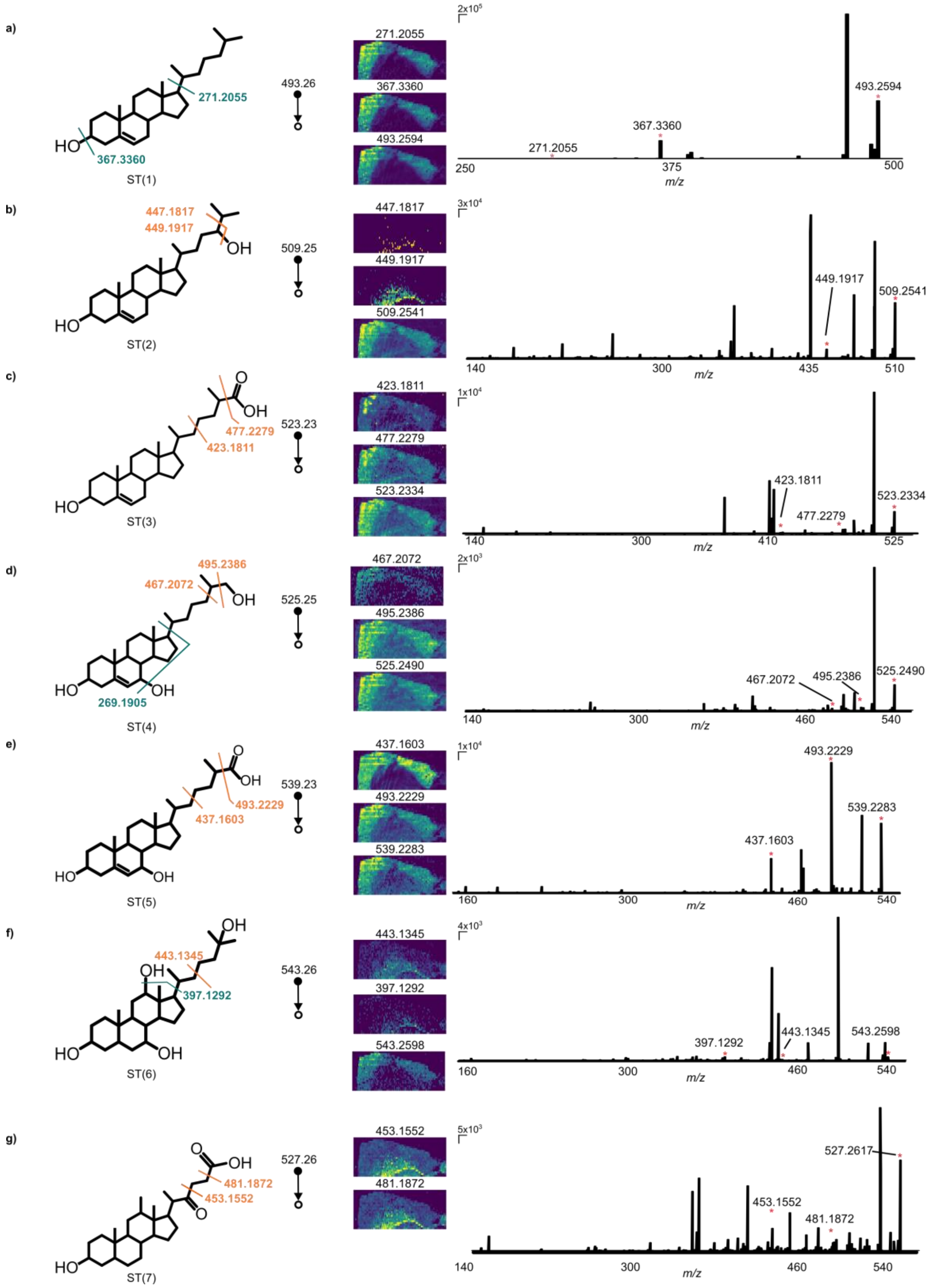


**Figure S17 Detected oxysterol species from human multiple sclerosis tissue by sequential FTMS$^2$I**

High mass resolution imaging of detected diagnostic product ions from cholesterol and cholesterol oxidation products (ST1-7) together with the respective fragmentation sites, precursor and product ion images, and FTMS$^2$ spectra are given in a-g. The details show that the mass channels of ST1, ST3, and ST4 have identical ion images for the product ions and the precursor ion (a, c-d), and the mass channels of ST2, ST5, and ST6 (b, e-f) may have multiple isomers. The abundance of the precursor ion for ST7 (g) is under the detection limit. All co-isolated precursor ions show different distributions (Fig. S15). For experimental details see Dataset 6 in Table S7.

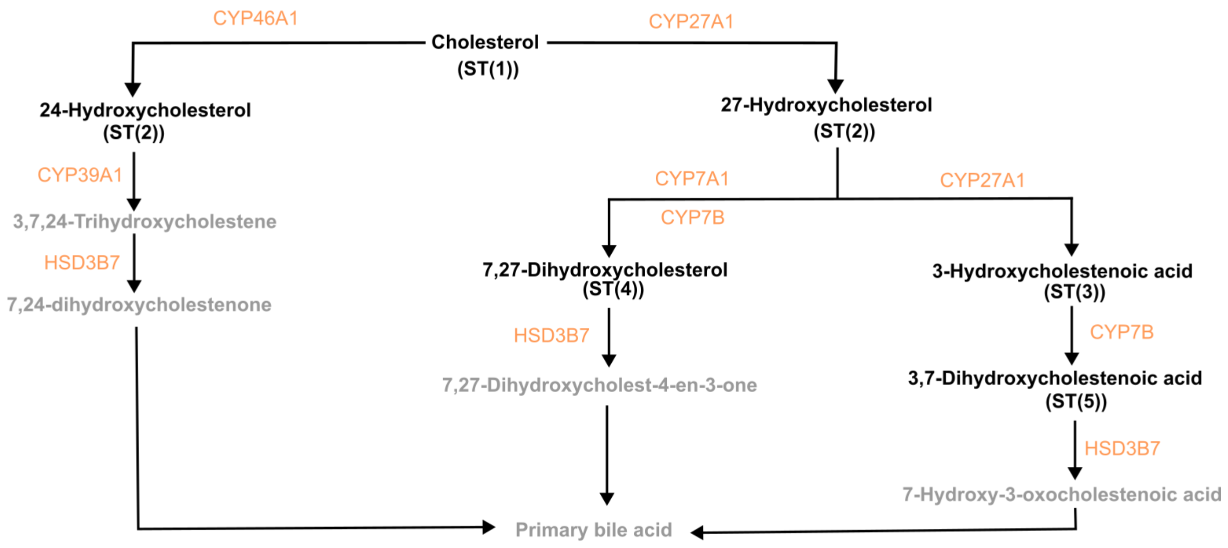


**Figure S18 Metabolic pathway for biosynthesis of bio acids from cholesterol**

The molecules highlighted in black with the abbreviations are detected in the human brain tissue, while the grey species are not detected. The responsible enzyme for each biochemical conversion is detailed in orange. The schematic is made from the KEGG database.

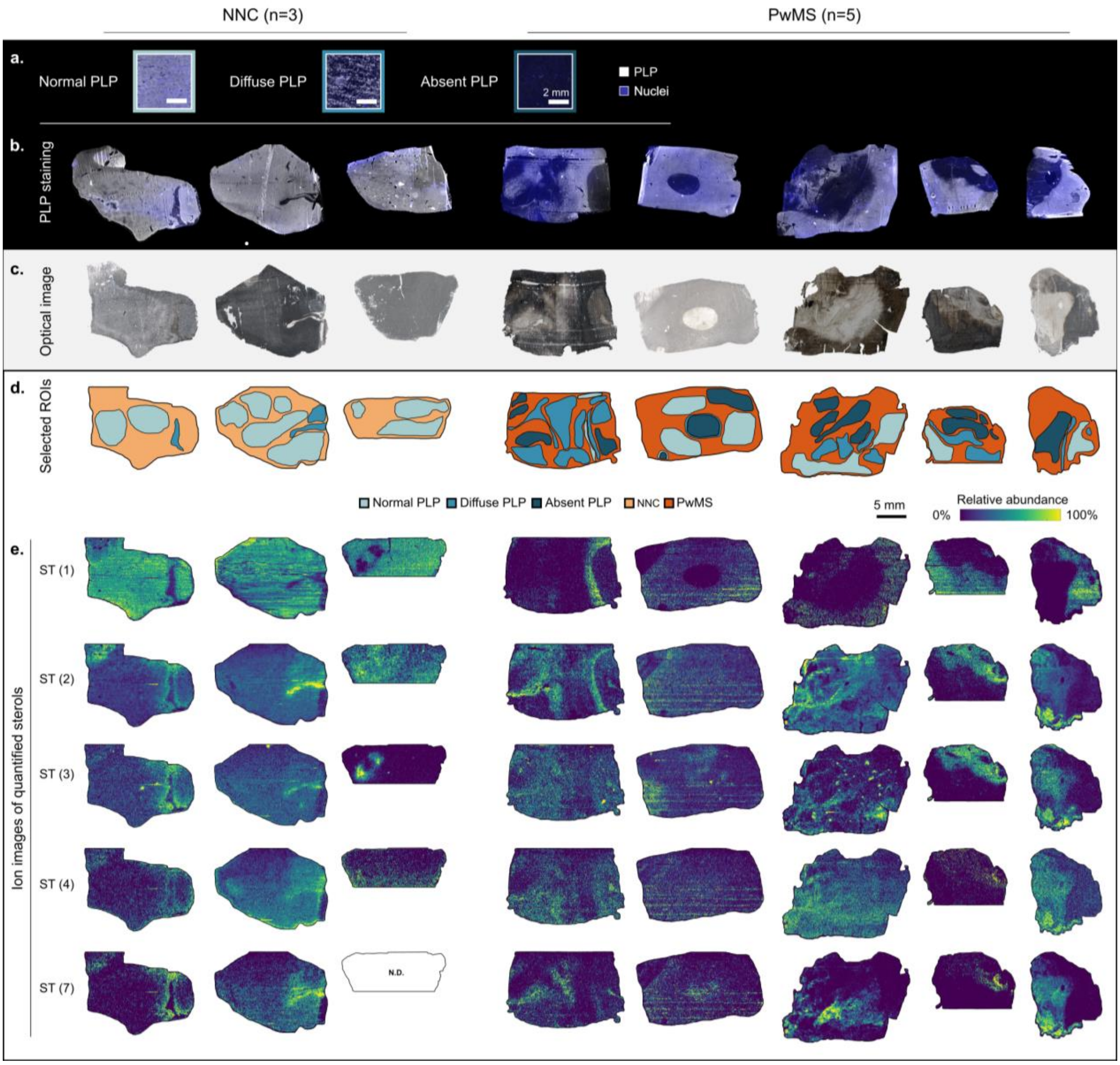


**Figure S19 Multimodal imaging of cholesterol and oxidized cholesterol products in human white matter brain tissue sections**

Representative classifier images of fluorescent proteolipid protein (PLP) immunoreactivity were acquired as stated in the Methods section. Scalebar is 2 mm. **(a)** Full tissue scans of PLP immunoreactivity of human brain tissues (NNC=3, PwMS=5). Image intensities are scaled individually. **(b)** Brightfield microscopy images acquired using a Meyer Instruments PathScan Enabler IV slide scanner device. **(c)** Resulting ROIs for each tissue section, colored according to the PLP classification (light blue: normal PLP, medium blue: diffuse PLP, dark blue: absent PLP). **(d)** Ion images of cholesterol and oxidized cholesterol products in human white matter brain tissues from NNC and PwMS normalized to TIC and the 99$^{th}$ percentile intensity. Scalebar is 5 mm and applies to images **(b)**-**(e)**. N.D.: not detected. Subsequent tissue sections are used for PLP and MSI imaging modalities. For experimental details see Dataset 5 in Table S10.

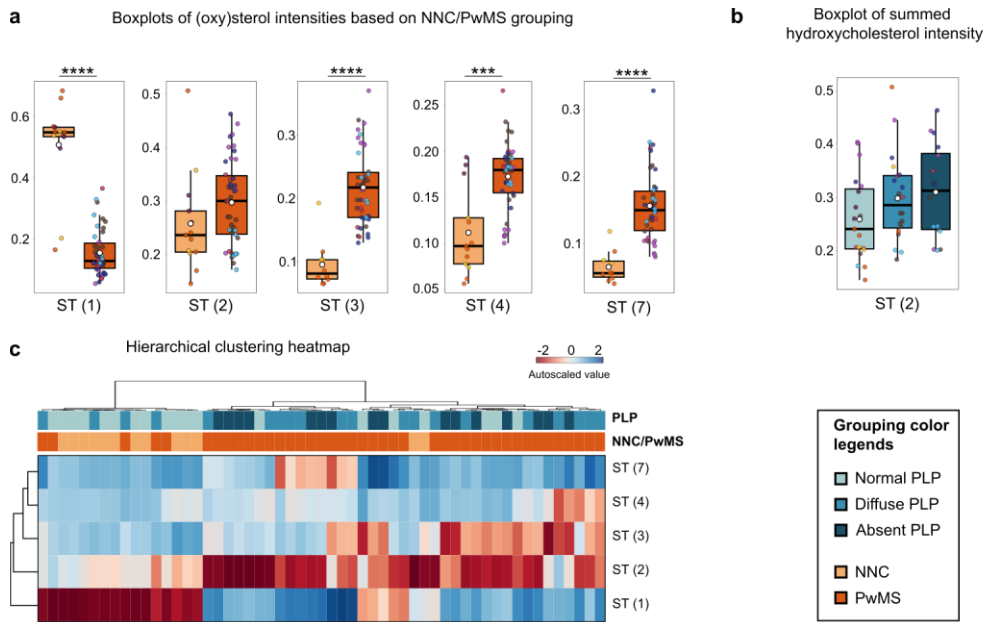


**Figure S20 Differentiation and clustering of brain tissue regions based on their oxysterol profiles**

**(a)** Boxplots representing the fractional abundance of detected sterols between ROIs corresponding to NNC (light blue) and PwMS (orange) subject samples. Note that since quantitation is based on $MS^1$, the two detected isomers 24-hydroxycholesterol and 27-hydroxycholesterol are denoted together as ST(2). All other presented mass channels are confirmed by $FTMS^2I$ to only contain the annotated sterol. Significant differences were observed for all the molecules except ST(2). To define significance, Wilcoxon test with Benjamini-Hochberg FDR correction was performed (*:p<0.05, **:p<0.01, ***:p<0.001, ****:p<0.0001). Coloring of points displays data from individual subjects as denoted by Table S1. **(b)** Boxplot representing the fractional abundance of ST(2) showing non-significant differences between ROIs corresponding to normal PLP (light blue), diffuse PLP (medium blue), and absent PLP (dark blue) subject samples. For additional boxplots, see Figure 5 in the main text. **(c)** Hierarchical clustering heatmap of (oxy)sterols across all ROIs shows a slight differentiation of normal PLP ROIs from diffuse and absent PLP ROIs and a considerable differentiation when the grouping is based on the subjects (NNC vs PwMS). For clustering, Ward method with Euclidian distance measures was used and values were autoscaled within each ROI sample. The coloring of hues for the autoscaled values is displayed in the top right corner. For experimental details see Dataset 5 in Table S7 and S10.

## References

(1) Lanekoff, I.; Heath, B. S.; Liyu, A.; Thomas, M.; Carson, J. P.; Laskin, J. Automated Platform for High-Resolution Tissue Imaging Using Nanospray Desorption Electrospray Ionization Mass Spectrometry. *Anal Chem* 2012, *84* (19), 8351–8356. https://doi.org/10.1021/ac301909a.

(2) Johan Lillja. Novel Strategies to Increase Throughput and Differentiate Lipid Isomers in Mass Specrometry Imaging. PhD Thesis, Uppsala University, Uppsala, 2023. id: diva2:1787714 (accessed 2025-04-10).

(3) Duncan, K. D.; Bergman, H. M.; Lanekoff, I. A Pneumatically Assisted Nanospray Desorption Electrospray Ionization Source for Increased Solvent Versatility and Enhanced Metabolite Detection from Tissue. *Analyst* 2017, *142* (18), 3424–3431. https://doi.org/10.1039/c7an00901a.

(4) Lillja, J.; Duncan, K. D.; Lanekoff, I. Ion-to-Image, I2i, a Mass Spectrometry Imaging Data Analysis Platform for Continuous Ionization Techniques. *Anal Chem* 2023, *95* (31), 11589–11595. https://doi.org/10.1021/acs.analchem.3c01615.

(5) Hansen, C. E.; Kamermans, A.; Mol, K.; Berve, K.; Rodriguez-Mogeda, C.; Fung, W. K.; van het Hof, B.; Fontijn, R. D.; van der Pol, S. M. A.; Michalick, L.; Kuebler, W. M.; Kenkhuis, B.; van Roon-Mom, W.; Liedtke, W.; Engelhardt, B.; Kooij, G.; Witte, M. E.; de Vries, H. E. Inflammation-Induced TRPV4 Channels Exacerbate Blood–Brain Barrier Dysfunction in Multiple Sclerosis. *J Neuroinflammation* 2024, *21* (1), 72. https://doi.org/10.1186/s12974-024-03069-9.

(6) Lassmann, H. Multiple Sclerosis Pathology. *Cold Spring Harb Perspect Med* 2018, *8* (3), a028936. https://doi.org/10.1101/cshperspect.a028936.

(7) Ihaka, R.; Gentleman, R. R: A Language for Data Analysis and Graphics. *Journal of Computational and Graphical Statistics* 1996, *5* (3), 299. https://doi.org/10.2307/1390807.

(8) Pang, Z.; Lu, Y.; Zhou, G.; Hui, F.; Xu, L.; Viau, C.; Spigelman, A. F.; MacDonald, P. E.; Wishart, D. S.; Li, S.; Xia, J. MetaboAnalyst 6.0: Towards a Unified Platform for Metabolomics Data Processing, Analysis and Interpretation. *Nucleic Acids Res* 2024, *52* (W1), W398–W406. https://doi.org/10.1093/nar/gkae253.
